# A systems thinking approach to understanding youth active recreation

**DOI:** 10.1186/s12966-022-01292-2

**Published:** 2022-05-12

**Authors:** Harriet Koorts, Paul M. Salmon, Christopher T. V. Swain, Samuel Cassar, David Strickland, Jo Salmon

**Affiliations:** 1 School of Exercise and Nutrition Sciences, Institute for Physical Activity and Nutrition (IPAN), Deakin University, 221 Burwood Highway, Burwood, Geelong, VIC 3125 Australia; 2 Centre for Human Factors and Sociotechnical Systems, University of the Sunshine Coast, Sunshine Coast, Queensland Australia; 3 Cancer Epidemiology Division, Cancer Council Victoria, Melbourne, Australia; 4 Sport and Recreation Victoria, Department of Jobs Precincts and Regions, Melbourne, Victoria Australia

**Keywords:** Physical activity, Active recreation, Health behaviour, Children, Adolescents, Systems approach, Systems modelling

## Abstract

**Background:**

Active recreation contributes to child and adolescent physical activity, however, factors affecting uptake are poorly understood at the systems level. The aims of this study were: (1) to use systems analysis methods to understand youth active recreation in Victoria, Australia, (ii) identify potential system leverage points to enhance active recreation, and (iii) explore stakeholder views of systems analysis methods for informing practice and policy decision-making.

**Methods:**

*Phase 1*: Umbrella review of systematic reviews (2013–2018), synthesising evidence for correlates, determinants and intervention evidence for promoting active recreation. *Phase 2*: Development of three systems models (ActorMap and two ActivMaps), depicting active recreation actors/organisations, correlates, determinants and intervention evidence. *Phase 3*: Development of causal loop diagrams (CLDs) and identification of leverage points based on the Action Scales Model. *Phase 4*: Model feedback via stakeholder interviews (*n* = 23; 16 organisations).

**Results:**

From the literature, 93 correlates and determinants, and 49 intervention strategies were associated with child and adolescent active recreation; the majority located at a social or individual level. Ten potential system leverage points were identified in the CLDs, which differed for pre-schoolers versus children and adolescents. Only time outdoors (an event leverage point) emerged for all age groups. Changes to the built and natural environment (i.e., land use planning, urban design) as a complete domain was a key structural leverage point for influencing active recreation in children and adolescents. Subject matter experts and stakeholder interviews identified 125 actors operating across seven hierarchical active recreation system levels in Victoria. Stakeholder interviews identified 12 areas for future consideration and recommendations for practice/policy influence.

**Conclusions:**

Our findings underscore the need for dynamic models of system behaviour in active recreation, and to capture stakeholder influence as more than a transactional role in evidence generation and use. Effective responses to youth inactivity require a network of interventions that target specific leverage points across the system. Our models illustrate areas that may have the greatest system-level impact, such as changes to the built and natural environment, and they provide a tool for policy, appraisal, advocacy, and decision-making within and outside of government.

**Supplementary Information:**

The online version contains supplementary material available at 10.1186/s12966-022-01292-2.

## Background

The physical and mental health benefits of physical activity are well-established [1, 2] and yet, in Australia, few children and adolescents are sufficiently active to achieve such health outcomes. Australia also has one of the lowest rates of physical activity at a population level, compared to the other 37 Organisation for Economic Co-operation and Development countries [3]. The Australian Health Survey (2011–12) showed that only 29% of children (5-11 years) and 8% of adolescents (12–17 years) achieved government recommended levels of physical activity for health (60 min of moderate-to-vigorous intensity physical activity [MVPA] per day) [4]. More recently, the National Health Survey (2017–18) reported that only 1.9% of 15–17 year olds met the physical activity guidelines [5]. Despite many interventions targeting youth physical activity, levels of inactivity have remained relatively unchanged over time and the effects of interventions targeting youth have been mixed [6]. Over a 15 year period, the overall prevalence of insufficient physical activity among Australian adolescents (11-17 years) was 87% in 2001, increasing slightly to 89% in 2016 [7].

Schools are recommended as a target setting for intervention [8], due to the considerable time children spend in this environment. Organised sport (e.g., community-based sports clubs), and active recreation (leisure-time physical activity undertaken outside of structured or organised activity [e.g. school physical education or active transport]) are also key targets for youth interventions, as this population often accumulate their physical activity via these activities [4]. Active recreation is defined as “leisure time physical activity undertaken outside of structured, competition sport. It is a set of activities within the wider range of physical activity options that also include active living, active transport and sport” (https://sport.vic.gov.au/our-work/participation/active-recreation#:~:text=Active%20recreation%20is%20leisure%20time,living%2C%20active%20transport%20and%20sport). Nonetheless, irrespective of the potential opportunities of these settings and activities; school-based interventions show only modest positive effects on physical activity [9] and organised sport contributes less than 4% of the variance in youth daily physical activity [10]. Whilst there is yet to be a consistent, sustained improvement in child and adolescent physical activity in Australia, non-organised physical activity (such as active recreation) may contributing to reducing the overall decline in youth activity levels [11].

Active recreation consists of a complex group of behaviours that are driven by multiple social, behavioural and environmental determinants [12–14]. These determinants are embedded within complex political and social systems, and no single intervention strategy is suitable at achieving sustainable long-term effectiveness [15]. More effective and sustainable interventions require comprehensive changes within multiple elements across many systems; that is, from a whole-systems perspective [16–18]. Changes to one domain (e.g., availability of active transport) within the ‘physical activity’ system may result in several changes or consequences in other domains that also impact physical activity [19], and these changes may be nonlinear and difficult to anticipate [20]*.* The lack of consistent positive effects of interventions has been attributed to many different factors (e.g., inconsistent intervention implementation in practice [21] and a lack of implementation theory or framework underpinning intervention design and delivery [22]). To date, there has also been an overemphasis on youth interventions that target individual level factors, without consideration of community or policy level factors [23]. Limitations of such reductionist thinking (i.e., studying a complex behaviour by reducing it to discrete variables) is not unique to physical activity. Linear, cause-and-effect approaches have been heavily criticised in many areas of health (e.g., road safety [24]), yet these reductionist approaches have dominated public health [17]. Notwithstanding the vast and ongoing contributions of *p*hysical activity research of the socio-ecological determinants underpinning active recreation behaviours; it remains unclear how these determinants interact [25] and how these interactions underpin the varying success of population level behaviour change approaches.

Systems approaches are fundamental for understanding complexity of health behaviours [26], and a systems approach is recommended when studying behaviours such as youth physical activity [27]. System approaches conceptualise physical activity as a product of the dynamics at play within several domains of influence [17, 20], and this approach can help elucidate why different outcomes exist when interventions are implemented in practice [28]. Systems analysis methods are commonly used to understand and depict the interactions between factors [29], including bi-directionality of their influence [28]. Popular systems analysis methods include ActorMaps, Accident Mapping technique (AcciMaps) [30] and causal loop diagrams (CLDs) [31]. ActorMaps are used to capture the connections and role of *influencers* in a given context (e.g., stakeholders). ActorMaps are a type of system map that depicts how individuals and organisations are interrelated in a system, and are useful for identifying opportunities to improve or intervene in the system via stakeholders. AcciMaps are an accident analysis method that is used to retrospectively analyse the multi-layered interactions between events, decisions and outcomes [30]. CLDs are used to pictorially demonstrate how different variables in a system are interrelated via positive and negative feedback loops, and provide a way to visualise the complex dynamics of a problem or behaviour [31]. They can also be used to identify where leverage points (places within a complex system where a small shift in one element can lead to large changes in others) [32] may exist; to inform development of physical activity promotion strategies.

The relationship between determinants of active recreation and subsequent intervention strategies has important policy implications in terms of which interventions governments and other physical activity stakeholders should target, and the complexity of approaches required to achieve population level change. Systems approaches can provide a starting point for stakeholders to develop well-informed health promotion strategies [33, 34], and methodologically, they are highly suited to studying active recreation given the breadth of organisations and activities involved. Systems approaches have been used successfully to study physical activity in children [35] and adults [36], to explore physical activity program planning and implementation with stakeholders [37], and in other, related, areas of public health prevention (e.g., incident causation for outdoor recreation in Australia [38, 39] and Australian physical activity policy [40]). Nonetheless, they have yet to be applied to model the youth ‘active recreation system’, and thus their useability and appropriateness among stakeholders working to promote active recreation is unknown. One of the many values of systems approaches, for example, is that systems models can provide a visualisation of the associations between variables (i.e., as shown in CLDs), however, this can be a double-edged sword. Depictions of complexity can act as a ‘tool’ for communication of complex issues [41], but they may be a barrier to translation for a broad audience. There is little evidence for the translatability of these methods among stakeholders working in active recreation. It is also unclear in physical activity, and more broadly in population health, if reductionist thinking has dominated the field due to a lack of knowledge of diverse areas such as political and environmental science (that are required for a deep understanding of complexity in health) [42]. Additionally, it is unclear if reductionist approaches are perceived more feasible in policy-making, as reductionist approaches do not necessarily incorporate the diversity of factors, determinants and contexts that underpin health outcomes [42]. These issues reflect major gaps in current knowledge surrounding the broader system of youth active recreation, and the utility of systems approaches among stakeholders working in the field.

The aims of this study were threefold. Firstly, to use systems analysis methods to understand the active recreation system for pre-schoolers, children and adolescents (herein referred to as children and adolescents) in Victoria, Australia. Secondly, to identify potential system leverage points using the Action Scales Model [43], and thirdly, to explore stakeholder views of these systems analysis methods as tools to inform practice and policy decision-making.

## Methods

**Fig. 1:**
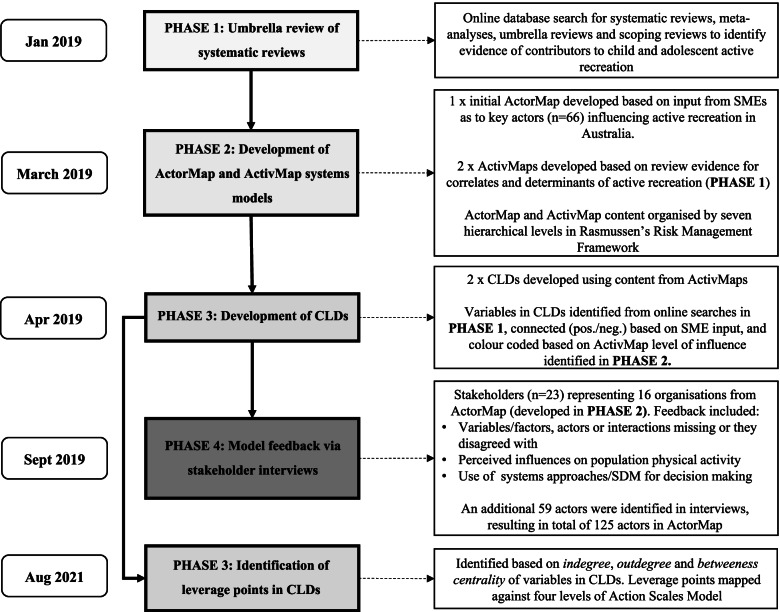
Study methodology. SME – Subject Matter Expert; CLD – Causal Loop Diagram; SDM – System Dynamic Model

The study involved four iterative phases. *Phase 1:* Umbrella review of systematic reviews to identify correlates, determinants, and intervention evidence for promoting active recreation among children and adolescents (January–February 2019). *Phase 2*: Development of ActorMap and ActivMap systems models to depict active recreation actors, correlates, determinants and intervention evidence; based on input from subject matter experts (ActorMap) and review findings (ActivMaps) (March 2019). *Phase 3*: Development of CLDs (April–August 2019) and identification of leverage points based on the Action Scales Model [43] (August 2021). *Phase 4*: Model feedback via stakeholder interviews (September 2019). Figure 1 presents an overview of the study phases.

### Phase 1: umbrella review of systematic reviews

An online literature search for systematic reviews and meta-analyses, umbrella reviews, and scoping reviews was conducted to identify evidence of contributors to child and adolescent active recreation. Differentiations between pre-schoolers, children and adolescents were based on definitions provided by published reviews. Children were defined in reviews as being in primary or elementary school and approximately 6 to 12 years of age. Adolescents were defined in reviews as being in secondary or middle/high school and approximately 13 to 18 years of age. Most reviews based their inclusion criteria on the mean age of participants in their studies. Seven online databases were searched (Medline, CINAHL, EMBASE, SPORTDiscus, Environment Complete, Urban Studies Abstracts, and PsychINFO), using a combination of free text terms and medical subject headings. Search strings were developed in consultation with a research librarian (Additional file 1). Reviews were eligible for inclusion if they had a primary outcome of increasing active recreation participation, were published in English between January 2013 to July 2018, and targeted children and adolescents. To ensure sufficient content and coverage of the literature; results included evidence outside of the Australian context. The search returned 5255 results. After removing duplicates and excluding titles and abstracts that clearly did not meet the inclusion criteria, 226 full texts were screened, resulting in 101 reviews for inclusion (seven meta-analyses and six umbrella reviews) (included reviews and data extracted listed in Additional file 2). Fifty-three reviews contained primarily observation studies and 48 reviews contained primarily intervention studies. Of the intervention evidence, 19 reviews did not provide sufficient information on individual intervention components. To address this, we retrieved additional information from 84 individual studies contained within these reviews.

### Phase 2: development of the ActorMap and ActivMap models

An initial ActorMap was drafted by three subject matter experts (HK, JS, DS and PS), to provide an overview of the actors (organisations, agencies, groups) that share responsibility for active recreation internationally, nationally and in Victoria. This is particularly important in Victoria, as promotion of active recreation is a key strategy to increase population physical activity, health and well-being. Approximately $1 billion AUD has been invested into the Victorian sport and active recreation system by the state government, since 2014 [44]. Subject matter experts were well placed to develop the initial ActorMap, due to their expertise in the project content area and methodology. Combined, subject matter experts DS and JS have over 30 years’ experience working with actors in the Victorian government active recreation sector, and HK & JS have over 30 years’ expertise in youth physical activity and active reaction (e.g. [10, 45–50]). Subject matter expert PS has more than 15 years’ of experience in applying ActorMap, AcciMap and CLDs (e.g. [51–55]). Actors were initially identified by subject matter experts listing all known actors relevant to active recreation based on their prior knowledge and expertise, which were then organised against the seven hierarchical levels of influence based on Rasmussen’s Risk Management Framework [56]; adapted for the Victorian context (Table 1). Levels included 1) International; 2) Government and government departments; 3) Regulatory and peak bodies, advocacy groups, and industry associations; 4) Local government, education, and sport; 5) Social environment; 6) Individual, and; 7) Built environment. Actors can have influence at multiple levels of the hierarchy, and so placement of the actors against the seven levels in the map was determined based on their *primary target* level of influence. For example, the World Health Organization (WHO) is placed at the ‘International’ level of the hierarchy as the WHO primarily targets global health issues, even though the WHO would also influence the second level of the hierarchy, ‘government and government departments’. The initial ActorMap was later refined during stakeholder interviews in Phase 4.

**Table 1:** Hierarchical levels influencing the Victorian active recreation system

Level	Definition
**International organisations**	Organisations based outside of Australia with an international membership, scope, or presence.
**Government and government departments**	Australian government organisations at the national and state levels responsible for the oversight and administration of specific government functions; at a state or national level
**Regulatory and peak bodies, advocacy groups, and industry associations**	A public authority or non-government organisation responsible for exercising autonomous authority over some area of physical activity in a regulatory or supervisory capacity; at a state and national level.
**Local government, education, sport, and health**	Organisations and groups tasked with serving their local community needs and directly or indirectly promoting active recreation; at a state level
**Social environment**	Family, friends, peers, and community that impact youth physical activity levels
**Individual**	Target population for active recreation
**Built and natural environment**	The surroundings or conditions that facilitate active recreation participation

Levels in column 1 are adapted from Rasmussen’s Risk Management Framework (RMF) [56]

Two AcciMaps (herein referred to as ‘ActivMap(s)’ as relevant to the physical activity/active recreation context of this study) were then drafted using results from the umbrella review (Phase 1), with factors also organised against the seven levels of Rasmussen’s Risk Management Framework (Table 1). Items were placed against hierarchical levels based on either (i) the level at which the stakeholder responsible resides (i.e., the factor ‘school policy to promote physical activity’ is placed at the level of ‘local government, education, sport and health’, as it is influenced by actors at this level), or (ii) the level at which the item relates (e.g., the factor ‘weather’ is placed at the level ‘built and natural environment’). Factors in the ActivMaps were connected colour coded to depict their relationship (positive, negative, null or mixed) with active recreation, according to the published literature.

### Phase 3: development of causal loop diagrams and identification of leverage points

Using the content from the two ActivMaps, two CLDs were produced using Kumu relationship mapping software (2019) (Retrieved from https://kumu.io/). The CLDs depict the variables influencing active recreation that were identified from literature searches (Phase 1), colour coded in the CLD based on the level of influence (Phase 2). Connections *between* variables in the CLDs illustrate the relationships between correlates and determinants (positive and negative), which we determined via the subject matter experts and stakeholder feedback (described in Phase 4). To identify potential leverage points, we used the Kumu software to identify CLD variables that were highly connected based on the ‘indegree’ (no. of inbound links), ‘outdegree’ (no. of outbound links) and ‘betweenness centrality’ (no. of times an element lies on the shortest path to another), and aligned them to the four levels of within the Action Scales Model [43]. The Action Scales Model is a conceptual tool for practitioners and policymakers to conceptualise, identify and appraise actions within a complex adaptive system [43]. The model draws on Meadows’ 12 “places to intervene” in a complex system, and extends the Intervention Level Framework [57] and Iceberg Model [58]. The Action Scales Model hierarchically categorises four levels to achieve system change, based on their degree of potential leverage (1. Beliefs [paradigm of how the system works], 2. Goals [goals of the system], 3. Structure [rules of the system], 4. Events [structure of the system]).

### Phase 4: model validation via stakeholder interviews

**Fig. 2:**
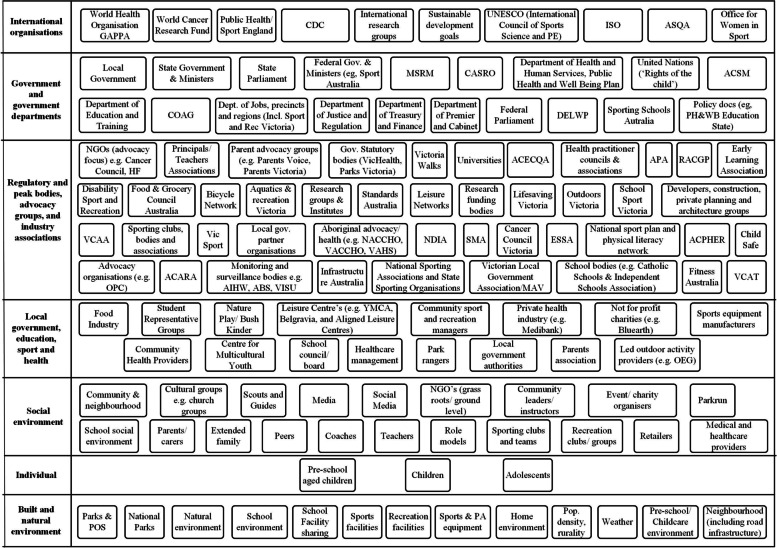
ActorMap of active recreation actors in Australia and internationally. PA = Physical Activity; POS = Public Open Spaces; PH&WBP = Public Health and Wellbeing Plan; PE = Physical Education; UNESCO = The United Nations Educational, Scientific and Cultural Organization; GAPPA = Global Action Plan on Physical Activity; ISO = International Sporting Organisation; ASQA Australian Skills Quality Authority; CDC = Centers for Disease Control and Preventions; ACHPER = Australian Council for Health Physical Education and Recreation; OEG = Outdoor Education Group; NGOs = Non-Government Organisations; AIHW = Australian Institute of Health and Welfare; APA = Australian Physiotherapy Association; ABS = Australian Bureau of Statistics; VCAT = Victorian Civil and Administrative Tribunal; VISU = Victorian Injury Surveillance Unit; NACCHO = National Aboriginal Community Controlled Health Organisation; MSRM = Meeting of Sport and Recreation Ministers; CASRO = Committee of Australian Sport and Recreation Officials; ACARA = Australian Curriculum, Assessment and Reporting Authority; DELWP = Department of Environment, Land, Water and Planning; ASM = American College of Sports Medicine; SMA = Sports Medicine Australia; NDIA = National Disability Insurance Agency; COAG = Council of Australian Governments; VACCHO = Victorian Aboriginal Community Controlled Health Organisation; VAHS = Victorian Aboriginal Health Service; ACECQA = Australian Children’s Education & Care Quality Authority; ESSA = Exercise and Sports Science Australia; MAV = Municipal Association of Victoria. RACGP = Royal Australian College of General Practitioners; VCAA = Victorian Curriculum and Assessment Authority; Red boxes – negative association with active recreation, green boxes – positive association with active recreation. Dashed lines - Preschool aged children, dotted lines – children/adolescents with a disability, solid black lines - typically developing children/adolescents

**Fig. 3:**
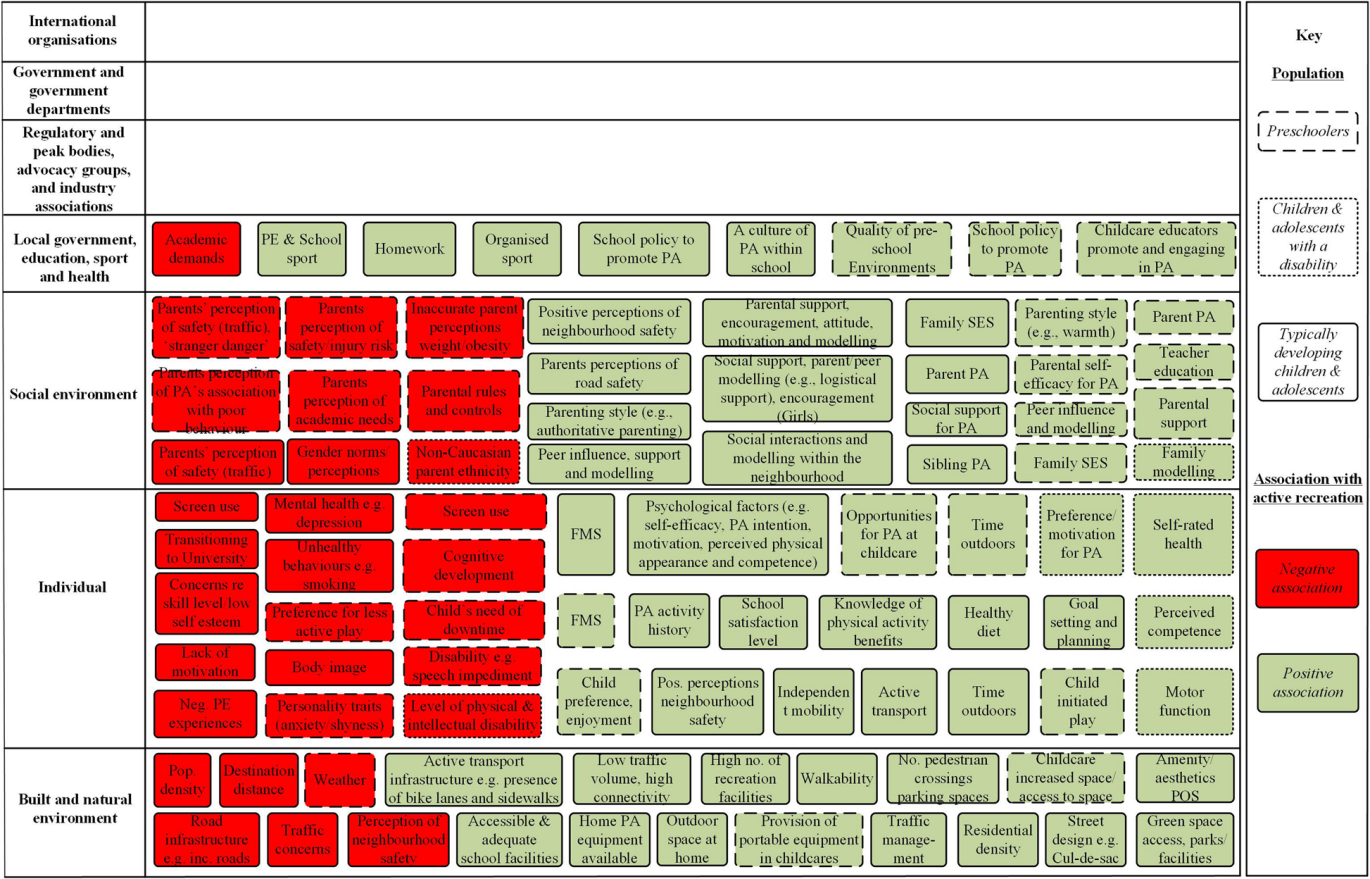
ActivMap of correlates and determinants of active recreation among pre-schoolers, children and adolescents. PA = Physical Activity, POS = Public Open Spaces, SES = Socioeconomic status, FMS = Fundamental Movement Skills, PE = Physical Education

**Fig. 4:**
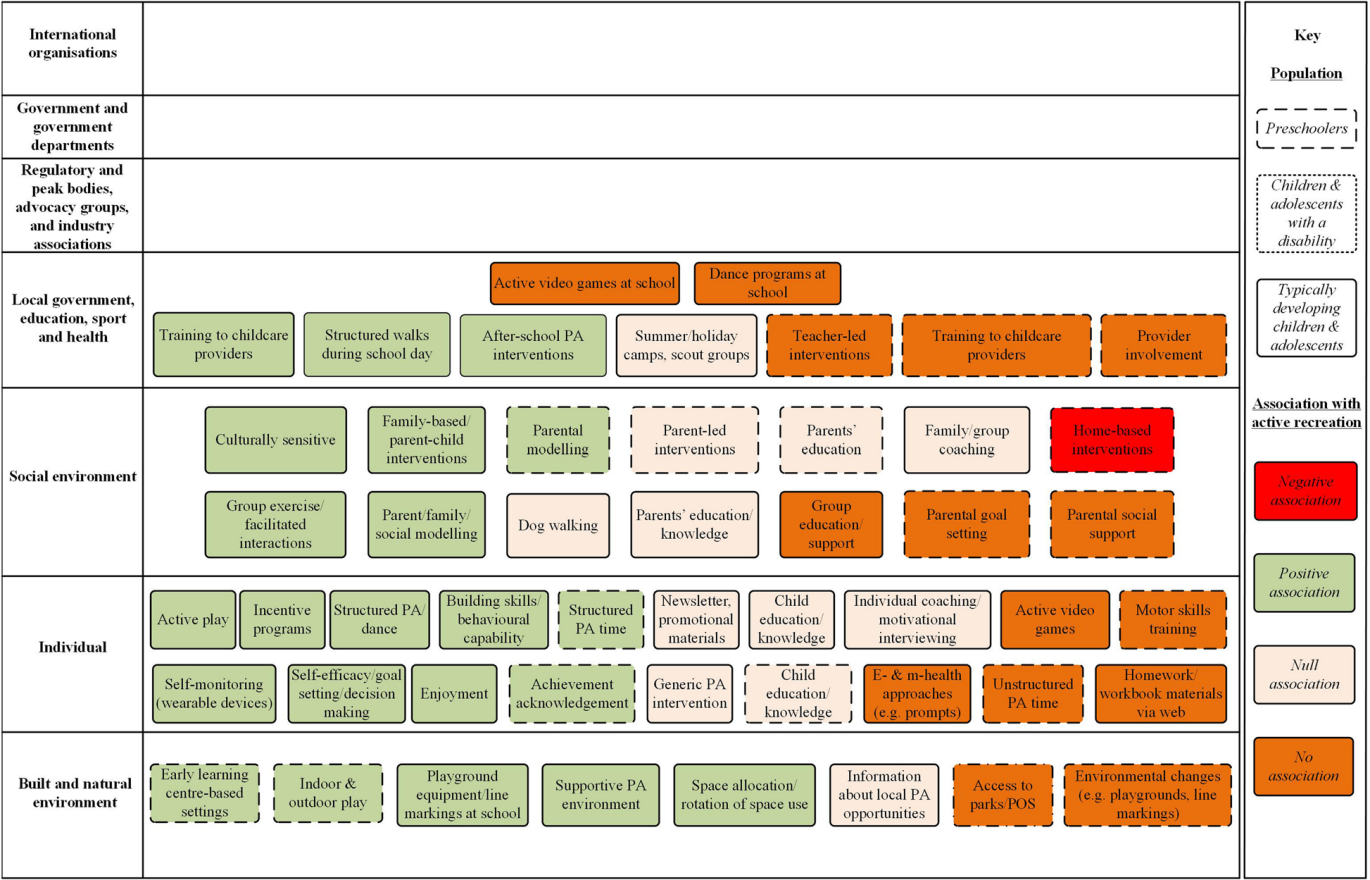
ActivMap of interventions to promote active recreation among pre-schoolers, children and adolescents. PA = Physical Activity, POS = Public Open Spaces

**Fig. 5:**
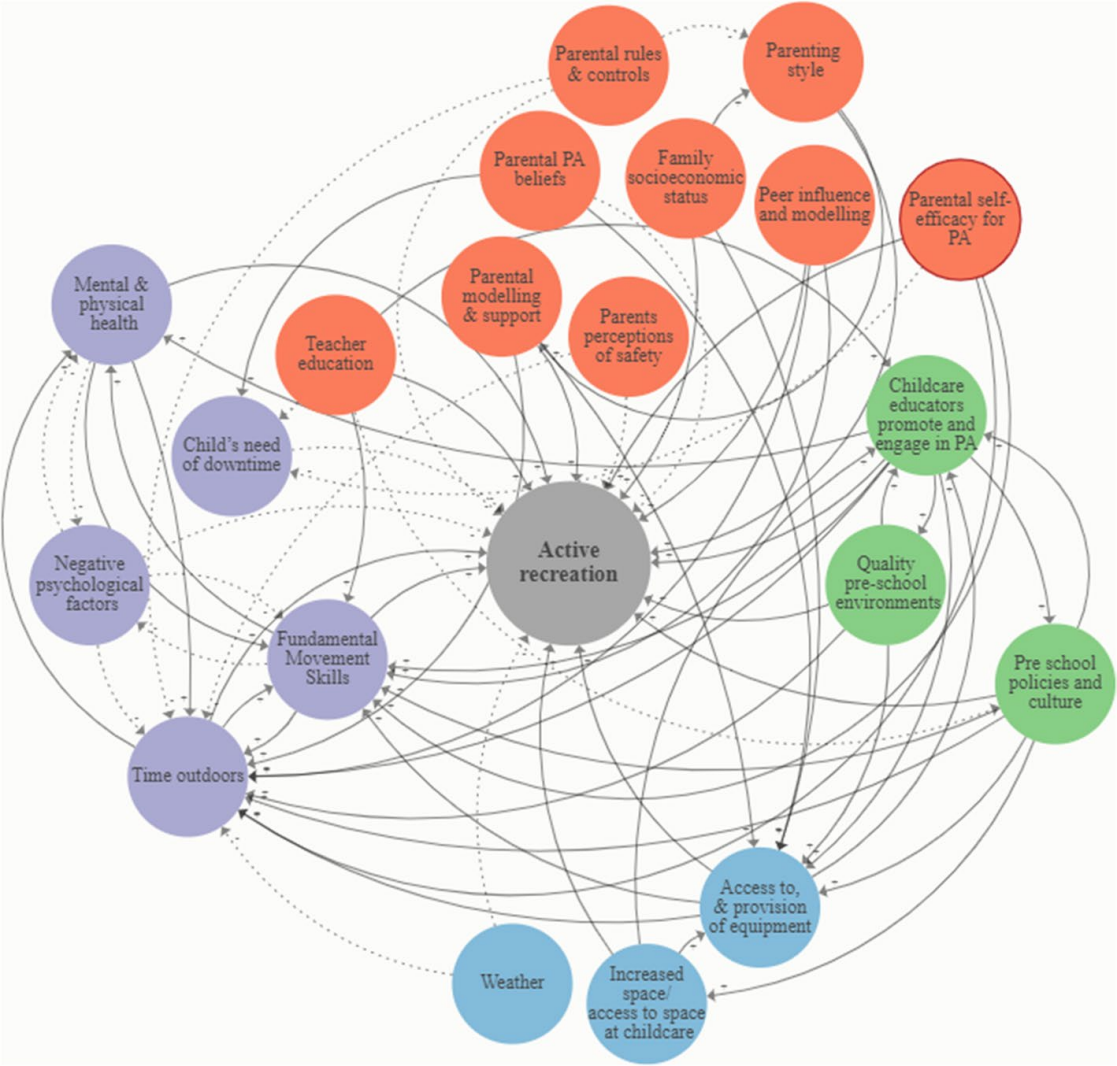
Causal loop diagram of correlates and determinants of active recreation among pre-schoolers. Solid black arrows (positive relationship), dotted black arrows (negative relationship). Purple (individual level); Blue (built and natural environment); Green (local government, education and health); Orange (social environment)

Stakeholder interviews were conducted to refine and validate all systems models (Figs. 1, 2, 3, 4 and 5). The ActorMap was used to identify key stakeholder organisations relevant to active recreation in Victoria that could be approached for interview. Organisations from the ActorMap that represented a cross-section of different types (e.g., government and non-government) and levels (e.g., state and national) were approached, and an opportunity sample of stakeholders (*n* = 23; representing 16 organisations) were recruited to participate in an online or in-person interview. Participants represented state and national government bodies, advocacy groups, as well as independent organisations, with expertise in health, planning, education, policy, sport, and disability. Interviews lasted between 60 and 90 min and were conducted by JS; CS and SC independently took notes. Interviews were conducted either online via Zoom teleconferencing software or face-to-face. The systems models were displayed on the screen during the interview, for participants to review and discuss. For face-to-face interviews, the maps were displayed on a screen and printouts of the maps were provided. The interviewer described the content of the maps, and explained the colour coding of the variables and arrows on display. All participants were asked: 1) if any *variables/factors/actors* in the maps were missing or that they disagreed with, 2) if any *interactions* in the maps were missing or that they disagreed with, 3) what they thought was most likely to influence population levels of physical activity, and 4) whether they would be likely to use systems approaches/a system dynamic modelling tool as part of future decision making. As the interview data comprised stakeholder feedback on visual aspects and content of the models, a structured interview script was not required. Formal qualitative analysis was therefore not appropriate in this instance (i.e., thematic analysis), rather, notes from interviews were summarised by CS and SC into ‘key areas’ for future consideration when using systems approaches for practice and policy decision-making. Stakeholders were invited to review and confirm their recorded feedback post interview.

## Results

### Active recreation ActorMap

Figure 2 presents an ActorMap containing 125 actors related to active recreation in Victoria. Sixty-six actors were initially identified by subject matter experts (HK, JS, DS and PS) and an additional 59 actors during stakeholder interviews. Actors were identified across all levels of the modified framework (Table 1), including: International (*n* = 10); Government and government departments (*n* = 19); Regulatory bodies, advocacy groups and industry associations (*n* = 44); Local government, education, sport and health (*n* = 16); Social environment (*n* = 20); Individual (*n* = 3); and, Built and natural environment (*n* = 13). A majority of the actors were located at the regulatory body/advocacy/industry group level (e.g., a government agency).

### Role, level and influence of active recreation actors (Fig. 2)

From interviews with stakeholders (*n* = 23), five key areas were identified that stakeholders felt either had not been captured in the ActorMap (Fig. 2) or was a limitation of the systems approach.

#### Key area 1: absence of additional sectors

Stakeholders discussed this in the context that other sectors may have a distal (i.e., international organisations such as the United Nations) or negative influence (i.e., food industry) on youth active recreation. Whilst stakeholders recognised that it was not the purpose of this study to capture these relationships, it was something they recommended is included for future research.

#### Key area 2: interactions between government levels

Engagement structures and coordinating mechanisms that existed between multiple levels of government were identified. For example, whilst local government had a more direct impact on ground-level delivery, there were government coordinating mechanisms at a state level - many of which were informal networks *within* government – that needed to be identified and depicted in Fig. 2, to reflect how governments connect across areas. As active recreation is inter-sectoral, a map of the ‘active recreation system’ needed to capture existing informal networks within government departments.

#### Key area 3: differentiating between the type and role of organisations

Based on the hierarchical levels used in Fig. 2, not-for-profit and grass roots organisations could be captured within the same level (i.e., Local government, education, sport and health). Stakeholders discussed that they differed and some would advocate ‘up’ and others would facilitate direct active recreation on the ground. In addition, statutory bodies have a degree of independence and thus should be a separate level, captured differently in terms of their role. The role and influence of actors could thus be very different, despite having an influence at the same ‘level’. The ActorMap methodology required that actors were depicted based on their organisational type and level of influence, whereas stakeholders requested to move actors to different levels based on how they interpreted their actual role and influence on active recreation in practice.

#### Key area 4: absence of grey literature underpinning models

Stakeholders discussed the many limits of relying only on the published peer-reviewed literature, and that systems models should include grey literature to capture activities ‘on the ground’. This was described as essential if the maps were to be useful in decision making, and that dynamic, as opposed to static, models would be an improved way of depicting the system. Stakeholders also discussed that policy documents (i.e., public health and wellbeing plans) should be reflected in the ActorMap as an influence, as they perceived this as something that influenced the system in the same way an ‘actor’ would.

#### Key area 5: absence of relational aspects between stakeholders

Example questions raised during interviews included “*to what extent do state and federal government care about active recreation?*” and “*is there policy alignment between different levels of government regarding this topic?*”. Critically, stakeholders were emphasising that responses to these questions reflect the nuances of policy influence on active recreation in Victoria, and these aren’t captured within the ActorMap. They described that not all actors are equal. Despite that the actors have been positioned in Fig. 2 ‘structurally’ based on the type of organisation, it was perceived that the ActorMap had not captured the relational aspects that influence active recreation and the political decision making that underpins government investment.

### Active recreation ActivMap

The literature search identified 93 correlates and determinants, and 49 intervention strategies, associated with child and adolescent active recreation. Literature identified included studies with typically developing children and adolescents, and children and adolescents with a disability. Figures 3 and 4, respectively, present ActivMap models depicting the correlates and determinants, and intervention strategies, associated with active recreation.

### Correlates and determinants of active recreation, and associated intervention strategies (Figs. 3 and 4)

For pre-schoolers, children and adolescents, correlates and determinants (*n* = 93) of active recreation (Fig. 3), related to hierarchical levels: local government, education, sport and health (*n* = 9), social environment (*n* = 28), individual (*n* = 35), and built and natural environment (*n* = 21). In total, 62 were positively associated with child and adolescent physical activity (coloured green) and 31 were negatively associated (coloured red) (Fig. 3). Most factors identified were related to the ‘social environment’, followed by factors at the ‘individual level’, compared to levels ‘built and natural environment’, and ‘local government education and health’. Of the four levels represented from Table 1, only variables related to local government, education and health were consistently positively related to pre-schoolers’ physical activity. Asides from ‘weather’, all other variables corresponding to the built and natural environment were also positively related to physical activity. More correlates and determinants were identified for typically developing children and adolescents (*n* = 56) than pre-school and early childhood aged children (*n* = 30) or children with a disability (*n* = 7).

Intervention strategies (*n* = 49) (Fig. 4) were located at the following hierarchical levels: local government, education, sport and health (*n* = 9), social environment (*n* = 13), individual (*n* = 19), and built and natural environment (*n* = 8). In total, 22 strategies led to improvements in physical activity (coloured green), 15 resulted in no change (coloured orange), 11 reported null results (coloured beige), and one was negative (coloured red) (Fig. 4). There were a greater number of intervention strategies reported for typically developing primary and secondary aged children (*n* = 30) than pre-school and early childhood aged children (*n* = 18). There were no active recreation intervention strategies from the umbrella review identified for children with a disability.

In both ActivMap models (Figs. 3 and 4), there was no literature evidence (correlates and determinants, or intervention strategies) identified relevant to levels corresponding to international organisations, government/government departments, and regulatory and peak bodies.

### Stakeholder reflections on ActivMap models (Figs. 3 and 4)

During interviews with stakeholders, overall, they were supportive about the use of systems models as a tool for decision-making, and they valued the visual representation of the spread of correlates and intervention strategies, including what has worked or not worked previously. So that we could capture differences in stakeholders’ feedback based on the correlates/determinants of active recreation (Fig. 3), versus intervention evidence for active recreation (Fig. 4); feedback was collated separately for each model. Seven key areas were discussed during interviews, relating to the breadth and coverage of the literature evidence, and interpretability of the ActivMaps. For Fig. 3, which depicted evidence for correlates and determinants of active recreation, four areas were identified.

#### Key area 1: evidence at the policy level

Stakeholders were surprised at the lack of evidence for active recreation relating to government, peak and advocacy bodies, and industry. This did not reflect their experience of influences in practice, for example, in terms of decision-making processes regarding the implementation of programs in the community, and funding for active recreation in policy documents.

#### Key area 2: absence of mediating factors

Whilst limitations of the literature base (lacking evidence for mediators) were acknowledged, stakeholders discussed that these factors were essential to depict a true systems model of active recreation. For example, variables, such as ‘access’, were insufficient in isolation and the nuance of factors mediating ‘access’ (such as family socioeconomic status) were required in order to capture the complexity of behaviour.

#### Key area 3: interpretability

Expanding definitions of the variables in the map was recommended to improve interpretability. Stakeholders discussed the challenges of the methodology capturing something complex and finding a balance between simplifying the information to retaining its richness. An interactive version of the map that allowed for an expansion of variables was suggested.

#### Key area 4: language and terminology

Stakeholders refined the language and terminology used in the map, to reflect language appropriate to the Victorian practice/policy context (i.e., naming Victorian specific parent advocacy groups).

For Fig. 4, which depicted intervention evidence for active recreation, three key areas were discussed.

#### Key area 1: strength of evidence

Strength of the evidence underpinning the intervention variables in Fig. 3 was questioned. This was in terms of transparency regarding the quality and quantity of data informing the model. For example, it was highlighted that ‘dog walking’ was reported to have a null effect on active recreation in Fig. 4, and yet this contradicted their experience and evidence of the positive benefits of dog walking. Stakeholders felt that a distinction needed to be clear over which variables were underpinned by one study versus multiple, as this has practice implications for interpretability.

#### Key area 2: applicability of the evidence

Stakeholders discussed this with particular reference to how the model captured rural and regional areas of Victoria. Some of the intervention evidence appeared relevant to primarily metro areas (i.e., correlates and interventions for the built environment). Whilst they acknowledged that the models depicted what was published in the literature, this raised discussion about the state-wide applicability of the models.

#### Key area 3: absence of local responses

Stakeholders provided many examples in their local jurisdiction that aligned with the intervention evidence in Fig. 4, but would not have been captured given the source of evidence underpinning the map was from published literature. Stakeholders referred to the need to capture local level evidence of interventions, in addition to the peer-reviewed literature.

### Active recreation causal loop diagrams

**Fig. 6:**
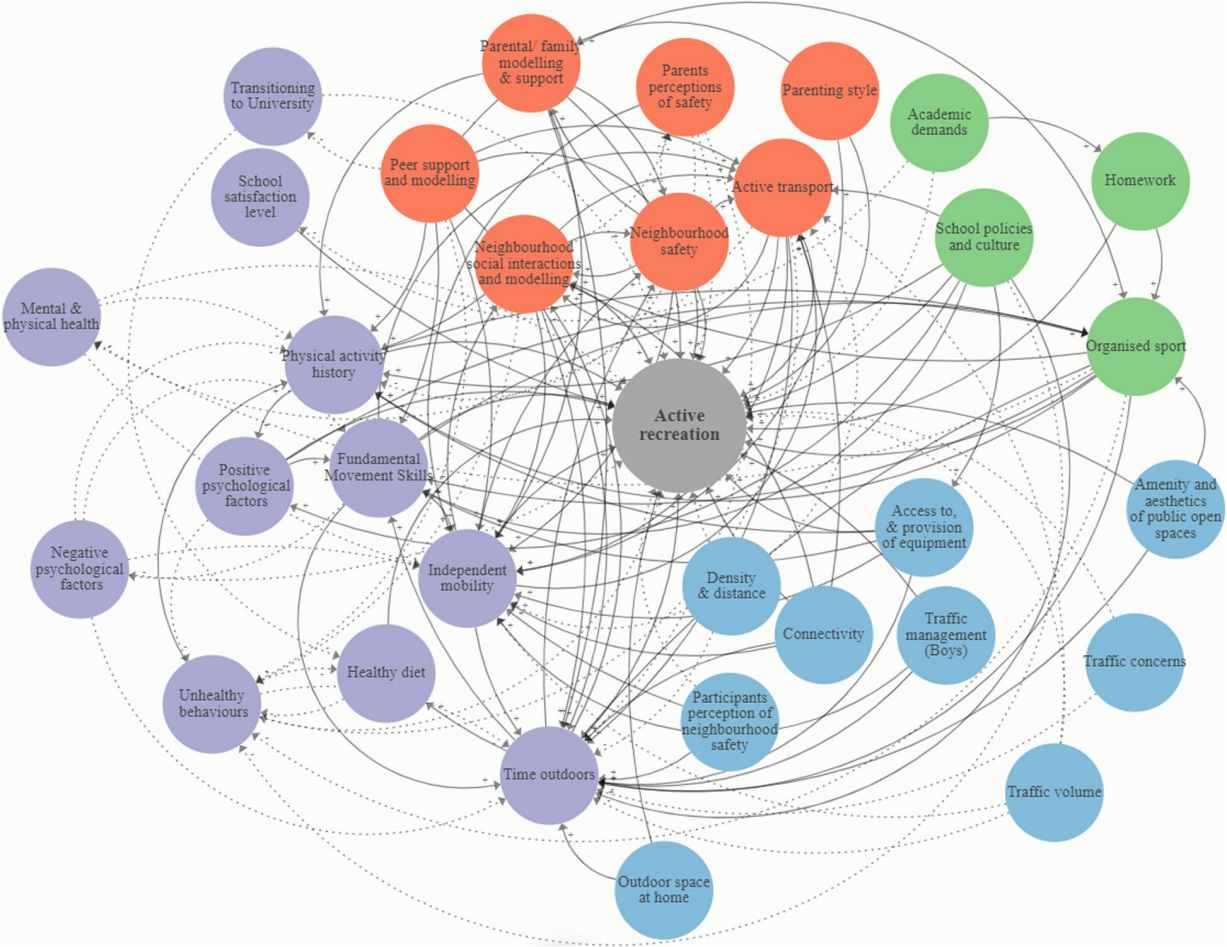
Causal loop diagram of correlates and determinants of active recreation among children and adolescents. Solid black arrows (positive relationship), dotted black arrows (negative relationship). Purple (individual level); Blue (built and natural environment); Green (local government, education and health); Orange (social environment)

Figures 5 and 6 present the CLDs of the correlates and determinants of active recreation among pre-schoolers, and children and adolescents, respectively. Directions of influence (positive and negative) in the CLDs was demonstrated using solid and dashed arrows.

The number of variables and their connectivity in the CLDs differed between review evidence for pre-schoolers (Fig. 5), and children and adolescents (Fig. 6). We identified domains corresponding to the ‘individual level’, ‘built and natural environment’, ‘local government, education and health’, and ‘social environment’. In the CLD of evidence for pre-schoolers (Fig. 5), there were a greater number of variables corresponding to the social environment compared to other domains. However, variables relating to the individual level were the most highly connected in the map (i.e., in terms of the absolute number of connections and the number of connections to other domains). In addition, almost all variables in Fig. 5 connected to at least one other variable from a different domain. Only the individual level variable ‘negative psychological factors’ was connected only to other variables at the individual level. In the CLD of correlates and determinants evidence for children and adolescents (Fig. 6), there were a greater number of variables corresponding to the individual level compared to other domains. Consistent with Fig. 5, only the individual level variable ‘negative psychological factors’ were unconnected to any other domain in the map. In Fig. 6, almost all variables corresponding to the built and natural environment were positively related to active recreation. In both CLDs, variables could have both a positive and negative (bidirectional) influence on other correlates and determinants, and active reaction.

### Leverage points influencing active recreation

The connectivity between variables in the CLDs (Figs. 5 and 6) differed greatly. In the CLD for children and adolescents (Fig. 6), most correlates and determinants were located at the individual level; the fewest were related to ‘local government education and health’. Variables related to the social environment were more often linked to individual level factors compared to the other levels within the map (Fig. 6). Variables were linked between all hierarchical levels, however, only one variable, related to the built and natural environment (amenity and aesthetics of public open spaces), was linked to local government education and health (organised sport). Most identified intervention strategies targeted the individual level (e.g., self-monitoring) and social environment (e.g., group education support). Eleven potential leverage points were identified from the CLDs (Figs. 5 and 6), relevant to active recreation among pre-schoolers (*n* = 5 leverage points) and children and adolescents (*n* = 6 leverage points). Leverage points corresponded to all four levels of the Action Scales Model [43] and to four of the seven levels of Rasmussen’s Risk Management Framework (leverage points: built and natural environment *n* = 1, individual level n = 6, social environment *n* = 3, and local government, education and health *n* = 1) (Table 2).

**Table 2:** Active recreation leverage points mapped to the Action Scales Model

Leverage point	Description^**a**^	Corresponding active recreation leverage point and influence level	
		Pre-schoolers (Fig. 4)	Children and adolescents (Fig. 5)
**Beliefs**	Deeply held beliefs, norms, attitudes and values of the individuals and organisations within the system. They are the foundations that cause the system to keep functioning as it does, and are reflected in the system goals.	**Individual**: Negative psychological factors (e.g.*, perceived self-worth and competence*)	**Social environment:** Peer support and modelling (e.g.*, family psycho-social setting*)
**Goals**	Goals, targets or ambitions that the system is working to achieve. Goals often drive the system to be structured as it is and therefore to work as it does.	**Local government, education, sport and health:** Childcare educators promote and engage in physical activity (e.g. *interventions delivered by teachers linked to increased physical activity)* **Individual:** Mental and physical health (e.g.*, sleep and diet facilitate play and activity*) **Individual:** Fundamental Movement Skills (FMS) (e.g.*, improvements in FMS can increase activity*)	**Individual**: Independent mobility (e.g.*, ability to engage in free-range activities without parental supervision*) **Social environment**: Neighbourhood social interactions and modelling (e.g.*, lack of children in neighbourhoods*)
**Structures**	Underlying structures and patterns that cause the events to occur. This includes the organisation of the system; the structures, information flows, processes and relationships between parts of the system.		^b^Built and natural environment (e.g.*, changes to playgrounds, urban design*) **Social environment:** Neighbourhood safety (e.g.*, traffic density*)
**Events**	Issues (behaviours and outcomes) that can be observed around us, and are symptoms of which arise from the system functioning as designed	**Individual:** Time outdoors (e.g.*, interventions located outdoors led to increases in physical activity*)	**Individual level:** Time outdoors (e.g.*, daily exposure to greenspace*)

*CLD* Causal Loop Diagram

^a^Descriptions in Column 2 from: Nobles, J. D., Radley, D., & Mytton, O. T. The Action Scales Model: A conceptual tool to identify key points for action within complex adaptive systems. *Perspectives in Public Health,* 2021

^b^Built and natural environment refers to the level of influence as opposed to a discrete variable

For example, a potential individual-level leverage point corresponding to ‘Events’ in the Action Scales Model, includes influencing youth time spent outdoors (Table 2). Among pre-schoolers, time outdoors can include interventions located outdoors encouraging increased physical activity, whereas among children and adolescents, time outdoors can include daily exposure to greenspace (Table 2). Time spent outdoors is classified as an ‘Event’ in the Action Scales Model, as it is a behaviour that results from how the system is designed. For example, youth time spent outdoors is an individual behaviour that is linked to the availability and access to outdoor recreation facilities and nature [59]. Availability and access to facilities in our environment is a direct outcome of how the system has been designed (e.g., urban planning and land use), and subsequently how it is used (e.g., time spent outdoors). The connectivity of this individual level variable (time spent outdoors) may mean that changes within the system that impact youth time spent outdoors, may lead to other, wider influences on within the system.

## Discussion

The purpose of this study was to explore the active recreation system in Victoria, Australia, using multiple systems analysis methods; ActorMap, ActivMap and CLDs. Feedback from key stakeholders was used to validate and refine all systems models developed in this study, providing key recommendations on ways these methods can be used in the future to assist with practice and policy decision-making. The current methods present a contribution that is distinct from a traditional evidence synthesis, and that applies systems analysis methods that have been used in safety (accident analysis) domains [30] to assess their utility for studying the complexity in youth active recreation. This macro-level approach enabled us to map how factors influencing active recreation and the actor(s) responsible, corresponded across multiple levels of influence in the system.

Despite calls for systems approaches to physical activity promotion [60], to our knowledge, this study is the first to combine multiple system models to understand youth active recreation. Our findings expose a disconnect between the literature evidence on factors influencing active recreation participation (correlates and determinants) and evidence for intervention strategies used to promote it. For example, it is recommended that effective action on youth active recreation requires an intersectoral, system-wide approach that enacts change in consultation with policymakers and stakeholders from multiple sectors [61]. Consistent with this recommendation, the ActorMap showed that actors influencing active recreation spanned all levels of the system. However, unlike what was depicted in the ActorMap, and what has been recommended for effective action on active recreation [61], the systematic review literature (depicted in the ActivMaps) corresponded to only four of the seven hierarchical levels. In addition, most of the known correlates, determinants and intervention strategies for active recreation were located at a social or individual level. This is despite growing evidence and global recommendations that actions to increase population activity require interventions targeting multiple sectors and settings [8, 62]. We found no literature review evidence for correlates or intervention strategies related to these government/industry levels, yet, almost three times the number of ‘influencers’ (actors) represented government and regulatory bodies/industry groups, than the social and individual level. This highlights the challenge of ecological models where the different levels of the system (e.g., ecological systems theory [63]) depict a ‘metaconcept’ but do not provide guidance on behaviour change at the individual level [64].

Our findings raise important questions regarding the real-world relevance of current evidence for youth active recreation with the broader active recreation system in practice. It reiterates the benefit of and need for systems approaches to changing physical activity environments and promoting active behaviours and policy actions [33], which include micro- through to macro-level strategies, and highlights the importance of grey literature and local-level data to inform development of contextually relevant system models that can benefit practice and policy. In physical activity, systems models (i.e., causal loop diagrams) demonstrate opportunities to implement policy actions across multiple areas of influence in the system, and systems approaches can help interpret the diverse relations between large numbers of factors, including their physical, commercial, sociocultural and political contexts [33]. Yet, historically in public health, the published evidence base has been skewed towards research that typically identifies simple, short-term, individual-level health outcomes and actions [17].

The CLDs demonstrate that determinants are not equal in their potential effect on the system. The connectivity between variables in the CLDs (Figs. 5 and 6) differed greatly, and thus the impacts of targeting one aspect related to active recreation over another is likely to vastly differ. This has important implications for deciphering potential leverage points for influencing active recreation. For example, almost all variables corresponding to the built and natural environment were positively related to active recreation, indicating that this may represent a potentially stronger leverage point for change than other parts of the system that had more limited connectivity. Despite that, this domain had fewer intervention strategies than those at a social or individual level, in the child and adolescent CLD (Fig. 6). ‘Access to and provision of equipment’, for example, was highly connected to variables at levels of the individual and local government, education and health, thus influencing many other variables in the system.

In contrast, although a child’s motor skill level is positively associated with active recreation (e.g. [65],), and interventions that target motor skills can lead to an increase in active recreation (e.g. [66],); motor skill level does not impact any other area of the system. In comparison to interventions targeting the built and natural environment, this domain appears a far weaker leverage point. As such, interventions targeting only motor skill development (individual level) may be less likely to achieve broader, sustainable shifts in behaviour, due to the other dynamic influences of the system (i.e., those related to the built and natural environment, such as access to and provision of physical activity equipment) that are not accounted for but still impact physical activity behaviours. Prior agent-based modelling supports this, and has shown that improving attitudes towards walking did not lead to sustained behaviour change without addressing other factors of the environment that were also conducive to walking [67].

Coverage of active recreation evidence also differed across population subgroups. More correlates and determinants were identified for typically developing children and adolescents, than pre-school and early childhood aged children or children with a disability, and there were no intervention strategies in the umbrella review identified for children with a disability. Strengths of a systems approach is that the needs of all groups, including vulnerable groups, are addressed for equitable health improvement. However, the implication of these findings is that these key actors (government/industry) and areas of influence related to active recreation remain ‘untapped’, as we lack evidence of their impact and how to intervene. Greater awareness of the complex interactions between the worldviews, perceptions and agendas of key stakeholders is considered necessary to increase impact and sustainability of population-based physical activity interventions [28]. Improved availability of policy evaluations may also address this gap in knowledge. However, the means by which Australian physical activity policies are monitored or reported is not always clear or mandated [68], and whilst the goals and beliefs of stakeholders can have greater influence on system behaviour, they are more difficult to change than proxy events (such as visible changes to our environment) [43].

Optimising those types of variables that have interconnectedness with others in a system, and targeting them as ‘leverage points’ (parts within a complex system whereby a small shift in one aspect can lead to significant changes in another [32]), may be one way of effectively changing system outcomes to achieve more sustainable impacts on broader population health. The rationale being that it enables examination of not only the direct effect of an intervention or exposure on active recreation, but also identifies the indirect effects on active recreation via wider system features. Our findings highlighted 10 potential leverage points for influencing youth active recreation, with the built and natural environment a key domain for influence. Leverage points corresponded to all levels of the Action Scales Model, however, their potential influence on system change differs. For example, according to the Action Scales Model, actions at the structure level (i.e., the built and natural environment) have a greater likelihood of leveraging systems change than those which are considered events [43]. The greatest potential leverage points are those relating to deeply held beliefs (i.e., negative psychological factors such as perceived competence), however, these are the most difficult to change at the population level. Our finding that the built and natural environment (e.g., changes to land use planning and urban design), was a potential structural leverage point for influencing active recreation, is consistent with global best buy investments to improve population physical activity [69]. Actions at this level have potentially more leverage for governments wishing to influence the active recreation system.

Stakeholder feedback was generally positive and supportive about the use of systems models as a tool to inform decision making. For example, despite the limited effectiveness of mass media campaigns at increasing population physical activity levels [70], many stakeholders acknowledged that this remained a common health promotion strategy. Nonetheless, a perceived limitation of the ActorMap was the absence of relational aspects between actors and the impact of interactions between organisations across hierarchical levels. Whilst not feasible in this study, other systems analysis methods are capable of analysing control and feedback relationships between actors at different levels. For example, Leveson’s Systems Theoretic Accident Model and Processes (STAMP) control structure method uses similar hierarchical levels to ActorMap but looks specifically at the control and feedback relationships between actors at different levels in the system hierarchy [71]. The volume of actors we identified (*n* = 125) highlights the breadth of influencers in the active recreation system, and potentially how challenging it may be to leverage or influence these different actors to achieve collective change or reduce duplication of effort. Understanding the power of actors and how they exercise it, has been shown to influence policy implementation [72] and systems change in obesity prevention approaches [73], and physical activity interventions scale-up [28].

Stakeholders also discussed that policy documents (i.e., public health and wellbeing plans) should be reflected in the ActorMap, as they perceived this as something that influenced the system in the same way an organisational or individual actor would. Whilst the inclusion of non-human actors (such as policy documents) in ActorMaps has previously been demonstrated [74], it was beyond the scope of this study to do so. Nonetheless, the lack of evidence for mediating factors was perceived as potentially limiting the models translatability into policy decisions. These findings are important for two reasons. Firstly, it raises questions regarding the most effective ways to capture different influences within systems models that reflect stakeholders’ own world views and perceptions, as well as that which is more ‘formal’ evidence, to ensure these maps meet the needs of intended users (stakeholders). Secondly, the desire for mediating relationships to be included in the maps reflects stakeholders’ needs for specifics and nuance to make evidence-based decisions in practice [75]. However, it also potentially risks amplifying the complexity of systems models that some stakeholders perceived would hinder effective research-practice-policy translation.

### Strengths and limitations

Major strengths of this study are the combined use of multiple systems approaches to understand active recreation, and involvement of stakeholders to refine the models for practice relevance and provide recommendations for future applicability. By including both published evidence for influences on active recreation, with the perspectives (‘mental models’) of key actors in the system; we were able to study the extent that research evidence translates to individuals’ experiences of practice.

However, the study is not without limitations. The scope of this project required that the data underpinning the models was based on physical activity literature published within a five-year period, which included an umbrella review of published systematic reviews. The review was conducted between January 2013 to July 2018 and there is the possibility that some evidence may have been missed due to our search parameters. For example, we found that dog walking had no relationship to active recreation based on the included review [76], and yet stakeholders’ noted that other research (published prior to January 2013 and thus would not have been captured through our search strategy), had shown positive associations [77]. In addition, a limitation of the ActivMaps is the inclusion of reviews that may have captured broader measures of physical activity, not just active recreation. Whilst the purpose of this study was not to appraise the literature as part of our review process, to promote rigour in our approach and capture any potential gaps in the evidence base, the models were developed using subject matter expert input and stakeholder consultation, in addition to the literature synthesis. Nonetheless, we cannot rule out the possibility of changes to the models had wider search parameters been feasible. Secondly, whilst we employed a two-stage process of identifying actors relevant to active recreation (via subject matter experts and stakeholders) to ensure comprehensiveness of the ActorMap, there is always the possibility of some actors being omitted, in particular as actors may change or emerge over time. Whilst this study aimed to understand the active recreation system in Victoria, Australia, to ensure sufficient content and coverage of the literature, results included evidence outside of the Australian context. This is consistent with current approaches to evidence synthesis that inform practice and policy decisions on physical activity, and reflects the types of data included in global physical activity recommendations (i.e., the World Health Organisation Global Action Plan for Physical Activity [8]). However, stakeholder feedback in this study emphasised the need for local level data and grey literature to inform the models and enhance contextual relevance, and that mediators of active recreation and nuances of policy influences between levels of actors needs to be captured. As the CLDs depict variables from published literature evidence, we were unable to modify them based on individual feedback regarding mediators. Whilst this study involved an iterative approach using several systems approaches that have been used elsewhere in public health research (e.g., smoking [34], obesity [78], and accident analysis [30]), these models are static and are unable to quantify the nature of relationships between factors or perform dynamic simulations. Whilst there are several examples of these systems approaches being used to address road traffic accidents [79] for example, there is no single accepted method for developing a systems model.

Future research, which incorporates evidence for the mediating and moderating relationships of factors within a dynamic systems model, including influences between actors (e.g., to support, inhibit or contribute to an agenda), would improve our understanding of active recreation and the potential outcomes of leveraging different parts of the system on population physical activity. Due to the timings of data collection, we were unable to obtain stakeholder feedback on the potential leverage points we identified. However, future research that captures stakeholder reflections on the relevance and appropriateness of leverage points, would potentially strengthen their translatability into practice. In addition, it was also beyond the scope of this project to incorporate the views of young people into the research process. Consulting with the target population has the potential to enhance the feasibility of active recreation strategies identified and inform the appropriateness of any recommendations; this should be a focus for future work. Our analysis boundaries meant we did not incorporate influences between actors across systems, or, for example, whether these influences were distal or negative. This created perceived limitations of the models among stakeholders. Models which incorporate this or include a simulation component (i.e., system dynamic modelling [SDM] [31]) would potentially provide greater insight into where and how to intervene across the multiple levels of the active recreation system, and help decision-makers identify the impacts of targeting one aspect of a system versus another [19, 28]. Advantages of SDM is that outcomes can enable identification of collaborative opportunities among actors, and reduced duplication of effort to enable strategic targeting of multiple parts of a system. Such models can also include a temporal component that can capture changes in influences over time [31] (e.g., changes in influences on active recreation and the time effects of different stakeholder actions on decision-making). The next steps in the development of such tools include the incorporation of evidence from grey literature (e.g. policy appraisal), performing quality appraisal on each individual evidence item, and holding a series of workshops with stakeholders to further develop each item and to address features of usability.

## Conclusion

Influences on child and youth active recreation participation are demonstrably complex and interrelated. Systems analysis methods offers a way to move beyond the cause-effect models that have been embedded, historically, in physical activity promotion research. Our findings underscore the need for dynamic models of system behaviour in active recreation, and the need to capture stakeholder influence as more than a transactional role in evidence generation and use. Multiple combinations of interacting factors influence active recreation participation in different contexts, with leverage points spanning all levels of system influence. Effective responses to youth inactivity require a network of interventions that target specific leverage points across the system. Our models illustrate areas that may have the greatest system-level impact, such as changes to the built and natural environment, and they provide a tool for policy, appraisal, advocacy, and decision-making within and outside of government.

## Supplementary Information

**Additional file 1.** Online databases and search strings.

**Additional file 2.** Reviews of correlates, determinants, and interventions for child and adolescent active recreation.

## Data Availability

The datasets used and/or analysed during the current study are available from the corresponding author on reasonable request.
