# Supplementary material for: A systems thinking approach to understanding youth active recreation
Source: Int J Behav Nutr Phys Act. 2022 May 12;19:53. doi: 10.1186/s12966-022-01292-2 (PMC9097093; doi:10.1186/s12966-022-01292-2)
Supplement: Supplementary file 1 — Additional file 1. Online databases and search strings. [file 12966_2022_1292_MOESM1_ESM.docx]

**Additional File 1.** **Online databases and search strings**

Via Medline, CINAHL, EMBASE, SPORTDiscus, Environment Complete, Urban Studies Abstracts, PsychINFO, using a combination of free text terms and medical subject headings. The search conducted on the 12^th^ September 2018 returned 5,255 results using the following search strings:

*Physical activity/active recreation string*

“physical* activ*” OR exercis* OR spORt* OR fitness OR recreation* “active play*” OR games OR walk* OR run* OR bicycl* OR bik* OR exertion OR “moderate activit*” OR “vigorous activit*” OR “leisure activit*” OR lifestyle OR participation OR leisure OR gym* OR swim* OR golf OR aerobic* OR tennis OR jogging OR yoga OR basketball OR football OR cricket OR “weight train*” OR “strength training” OR “resistance training” OR bowls OR danc* OR exercise bike OR treadmill

*Correlate/determinant string*

correlates OR determinants OR intrapersonal OR interpersonal OR organisational OR biological OR psychological OR “emotional fact OR*” OR “demographic fact OR*” OR “physical environment*” OR “built environment*” OR “natural environment*” OR “social environment” OR “community design” OR neighborhood OR neighbourhood OR “urban planning” OR “urban form” OR “urban design” OR walkability OR connectivity OR access* OR facilities park OR “green*space” OR “open space” OR greenway OR “urban regen*” OR safety OR incivilit* OR infrastructure OR pedestrian OR “road environment” OR path sidewalk* OR crossing* OR trail* OR “transport system*” OR “health promotion*” OR “primary prevention*” OR “preventive medicine*” OR “public health*” OR “health education*” OR prevent* OR promot* OR program* OR project* OR educat* OR campaign* OR intervent* OR strategy* OR polic* OR breaktime OR “break time” OR “school recess” OR recess OR playtime OR “lunch time” OR lunchtime OR “free play” OR “schoolyard” OR “playthings” OR “outdoorspace*” OR “playground*” OR “playground facilit*” OR “playground equip*” OR “active playground*” OR after-school OR “after school” OR afternoon OR evening OR “critical window” OR “critical hours” OR “out of school” OR “outside of school” OR “before school”

*Demographic string*

child OR infant* OR youth* OR youn* OR adolesce* OR teen young adult* OR “school age” OR boys OR girls

*Review string*

Review* OR meta-analys* OR cochrane OR “rapid evidence assessment” OR summary OR research synthesis
