# Supplementary material for: A systems thinking approach to understanding youth active recreation
Source: Int J Behav Nutr Phys Act. 2022 May 12;19:53. doi: 10.1186/s12966-022-01292-2 (PMC9097093; doi:10.1186/s12966-022-01292-2)
Supplement: Supplementary file 2 — Additional file 2. Reviews of correlates, determinants, and interventions for child and adolescent active recreation. [file 12966_2022_1292_MOESM2_ESM.docx]

**Additional File 2: Reviews of correlates, determinants, and interventions for child and adolescent active recreation**

| **#** | **Authors** | **Purpose** | **Sample** | **Review type; Study N** | **Brief findings** |
| --- | --- | --- | --- | --- | --- |
| 1 | An, Yang (1) | Quantify the influence of neighbourhood safety on childhood obesity risks. | Aged ≤ 17 years | SR and MA; 22. studies | Living in unsafe neighbourhoods was associated with a reduction in PA duration by 0.13 (95%CI = 0.06, 0.19) h week^-1^ for children and adolescents. |
| 2 | Aura, Sormunen (2) | Identify and describe adolescents’ health-related behaviours from a socio-ecological perspective. | Aged 11 – 18 years | SR; 90 studies | Positive association with PA for family wealth, parental education, parent support and modelling, school satisfaction, peer group, and environment comfort. Negative association with PA for academic demands, self-esteem, ethnicity, age, and BMI. Some variation according to sex. |
| 3 | Ball, Carver (3) | Highlight promising approaches for promoting PA and reducing sedentary behaviours | Not limited. | Rapid review | Limited evidence overall and complexity inherent. But some promise with community approaches, local and state government, provision of parks, peer programmes, and motivational programs. |
| 4 | Biddle, Braithwaite (4) | Can interventions increase PA in girls? | Pre-adolescent females. | SR and MA; 22 studies | Shorter PA participation or education interventions have a small positive effect. Atheoretical interventions more effective. |
| 5 | Bonell, Wells (5) | Examine effectiveness of school-environment interventions | School aged children | SR; 10 studies | Interventions enabling students to advocate for changes in physical-activity environments have some benefit |
| 6 | Borde, Smith (6) | Determine the impact of school-based interventions on objectively measured PA | Adolescents | SR and MA; 13 studies | School based interventions did not increase PA. Intervention characteristics did not moderate the effect. |
| 7 | Brown, Hume (7) | Review the efficacy of PA interventions on PA and examine potential mediators | Aged 5 – 12 years | SR; 31 studies | Mediators included cognitive/ psychological factors, social factors, but not built environment. |
| 8 | Brown, Atkin (8) | To examine family interventions on child PA | Children | SR and MA; 47 studies | Family interventions increased PA. Goal setting, reinforcement, development of movement skills improved PA. Education alone did not. |
| 9 | Bush and Garcia Bengoechea (9) | Describe what is known about interventions to increase PA. | Adolescents | Mapping review; 46 studies | Intervention settings include interpersonal, social network, community, organisation, and policy domains. The authors presented limited evidence for each domain. |
| 10 | Carlin, Murphy (10) | Determine whether interventions to increase walking work | Children and adolescents | SR; 12 studies | Interventions can increase walking or PA. Positive findings for waking school bus and multicomponent interventions, contrasting findings for education interventions. |
| 11 | Cerruti Dellert and Johnson (11) | Examine the effect of interventions delivered to parents and children on children's PA and BMI | Children | MA; 21 studies | Interventions with children and parent can have a moderate effect on child PA. Interventions targeting children alone do not increase PA. |
| 12 | Chung, Ersig (12) | Examine the contribution of peer factors to diet and exercise of adolescents. | Aged 13–19 years | SR; 32 studies | Positive associations for peer exercise and PA. Influenced by closeness of friendship. May be moderated by sex. |
| 13 | Condello, Puggina (13) | To provide an overview of behavioural determinants of PA | Entire lifespan | Umbrella review; 17 reviews | Positive associations with PA for previous PA, school sport participation (adolescents), mobility, and homework. No association for sport participation (children) or diet. Negative associations for screen time and vigorous PA (with overall PA). |
| 14 | Corepal, Tully (14) | Investigate the effectiveness of controlled trials using behavioural incentive interventions | Aged 5 – 18 years | SR and MA; 22 studies. | Limited evidence for behavioural incentives. |
| 15 | Cortis, Puggina (15) | To give an overview of psychological determinants of PA | Entire lifespan | Umbrella review; 20 reviews | Positive associations for intention, knowledge of benefit, self-efficacy, goal setting, motivation and self-worth, and perceived competence, appearance, or fitness. |
| 16 | Craike, Wiesner (16) | Examine the effectiveness of interventions to improve PA and determine the characteristics of effective interventions | Lifespan | Umbrella review; 17 reviews | Mixed effectiveness for all interventions. Interventions in the community, targeting parents, or multi-component interventions more likely to be effective. |
| 17 | Crawford, Stafford (17) | Examine strategies that facilitate inclusion in play among children with physical disabilities in childcare centres | Children with physical disabilities | SR; 9 studies | Adult facilitator and environment factors may be positive for PA. |
| 18 | Cushing, Brannon (18) | Evaluate and quantify the evidence for health promotion interventions | Children and adolescents | SR and MA; 89 studies | Interventions that targeted individuals, families, or schools with media can increase PA. |
| 19 | Direito, Carraca (19) | Examine the effectiveness of mHealth interventions on PA | Aged < 19 years | SR and MA; 21 studies | mHealth interventions did not significantly increase PA relative to the comparison. |
| 20 | Dobbins, Husson (20) | Examine school-based PA programs for promoting  PA and fitness | Children and adolescents aged 6 – 18 years | Cochrane review; 26 studies | Some evidence that school-based PA interventions had a positive impact on PA. |
| 21 | Draper, Grobler (21) | To determine the impact of social norms and social support on PA in adolescents | Adolescents | SR/ Scoping review of reviews; 30 reviews | Positive associations with PA for family influences (e.g., income, support, sibling activity), friend support and activity, and neighbourhood level interactions. Negative associations for bad past experiences (e.g., PE class) and perceived gender norms. |
| 22 | Errisuriz, Golaszewski (22) | Examine physical education interventions effect on PA | School children | SR; 12 studies | PE interventions can increase PA during class but do not seem to benefit leisure time PA. |
| 23 | Erwin, Ickes (23) | Examine the effect and characteristics of recess interventions on PA | Children | SR and MA; 13 studies | Recess interventions have a positive effect on child PA. These are more effective in younger children. Longer recess duration, more play equipment, structured and semi structured play, and teacher involvement can be beneficial. |
| 24 | Escalante, Garcia-Hermoso (24) | Examine playground design interventions for PA | Children | SR; 8 studies | Inconsistent findings. Some interventions such as playground markings and game equipment did increase PA in some but not all groups studied. |
| 25 | Figueroa and An (25) | Examine the relationship between motor skill competence and PA among pre-schoolers | Pre-schoolers aged 3-5 years. | SR; 11 | Positive association between motor skills and PA. May be mediated by gender. May interact with activity type. |
| 26 | Fisher, Smith (26) | Examine genetic and environmental influences on  objectively measured PA | Children | SR; 7 twin studies | Modest influence of genes on childhood PA. In everyday life environment a stronger determinant. |
| 27 | Gordon, Tucker (27) | Examine the effectiveness of PA interventions on PA participation | Pre-schoolers | MA; 15 studies | Overall small to moderate intervention benefit. Greater benefit in shorter interventions, teacher led interventions, early learning setting or outdoor interventions, or interventions that involved environmental change. Home interventions negative. |
| 28 | Herring, Sailors (28) | To examine evidence for genetic determinates of PA | Humans and animals | NR | Neural signalling and pleasure/reward systems in the brain may positively contribute to PA |
| 29 | Hesketh, O'Malley (29) | Synthesise evidence for determinants of change in PA | Children aged 0 – 6 years. | SR; 44 studies | Determinants of PA included sex, motor skills, child knowledge, parental factors, goal setting, provider support/ training, PA opportunity, use of portable equipment, and supply of curriculum materials. |
| 30 | Hesketh, Lakshman (30) | Synthesise the qualitative evidence of barriers and facilitators to activity behaviours in preschool-aged children | Parents and pre-school aged children | SR; 43 studies | Perceived facilitators include sex (male), facilities, enjoyment, active parents, and peers. Perceived barriers include perceptions of safety, need for down time, and weather |
| 31 | Hieftje, Edelman (31) | Examine evidence for electronic media-based interventions on health and safety behaviour change | Children and adolescents | SR; 19 studies | Some suggestion of increase in PA in small number of studies. |
| 32 | Hodges, Smith (32) | Identify common factors associated with the promotion or hindrance of PA in young children | Children aged 2-5 years | SR | Pre-school quality and amount of play equipment positively associated with PA. Barriers include child personality (e.g., shyness), innate preference, disability, and parent perceptions. |
| 33 | Hynynen, van Stralen (33) | Evaluate the effectiveness and characteristics of school-based interventions to increase PA and decrease sedentary behaviour | Adolescents aged 15 – 19 years | SR; 10 studies | Small to medium sized increase in PA following intervention. Effectiveness was unrelated to length of intervention or mode of delivery. |
| 34 | Jaeschke, Steinbrecher (34) | Provide an overview of socio-cultural determinants of PA. | Entire lifespan | Umbrella review; 23 reviews included. | No socio-cultural determinants had convincing evidence for PA. Family structure, encouragement, and companionship may be positively associated with PA. Parental modelling does not appear to be associated with PA. |
| 35 | Jiying, Robbins (35) | Examine the effect of interventions on objectively measured PA | Pre-school children aged 2-5 years | SR; 24 studies | Less than half of the interventions increased PA or decreased sedentary behaviour. Centre based, theory-driven, multicomponent interventions that included a structured PA and targeted both parents and their children appeared more effective. |
| 36 | Johnstone, Hughes (36) | Determine the effect of active play interventions on PA and movement skills | Children | SR and MA; 4 studies (2 in meta-analysis) | No significant effect for active play interventions on children’s PA. The authors read both studies. |
| 37 | Kader, Sundblom (37) | Review the effectiveness of parental support interventions on dietary habits, PA, or overweight and obesity | Children aged 2 – 18 years | SR; 35 studies | Limited evidence for the effectiveness of  parental interventions to increase PA in children. |
| 38 | Kaushal and Rhodes (38) | Examine how the how the home physical environment relates to adult and child PA | Children | SR; 49 studies | Home PA equipment, neighbourhood safety, and exergames associated positively with PA. |
| 39 | Lai, Costigan (39) | Determine whether school-based interventions focusing on PA, fitness, or movement competency produce a sustained impact in these outcomes. | Children and adolescents | SR; 14 studies | Majority of interventions increased some measure of PA. These included theory based interventions, interventions that utilised education, and combination interventions. |
| 40 | Laird, Fawkner (40) | To map the associations and to perform meta-analyses on types of social support for adolescent girls’ PA. | Adolescent females | SR and MA; 84 studies | Positive evidence for social support from all sources apart from teachers, emotional support including encouragement, modelling from mother, and instrumental support/ facilitation (e.g., transport, logistics, financial). Age and location moderators. |
| 41 | Langford, Bonell (41) | Assess evidence for the effectiveness of the health promoting school’s framework for improving the health and well-being of students | Children and adolescents aged 4-18 years. | Cochrane review; 67 studies | Small increases in PA on average. |
| 42 | Larouche (42) | Summarise built environment correlates of cycling among school-aged children. | School aged children | SR; 12 studies | Safety, recreation and pedestrian facilities, and connectivity positively impact cycling. Larger population, greater distance, and safety negatively impact cycling. |
| 43 | Larouche, Mammen (43) | Examine the effectiveness of active school transport interventions | Children and adolescents | SR; 27 studies | Mixed findings for increasing active school transport. Longer follow up, small school size, higher school socioeconomic status, and safer routes were associated with better outcomes. |
| 44 | Larouche, Mammen (43) | Association between active school transport and daily PA | School children aged 5.0–17.9 years old. | SR; 73 studies | Active school transport positively associated with daily PA. |
| 45 | LeBlanc, Chaput (44) | Examine the relationship between active video games and health indicators | Aged 0 – 17 years | SR; 52 studies | Active video games positively correlated with PA but interventions with active video games do not increase PA. |
| 46 | Lee, Tamminen (45) | To review the qualitative research examining determinants of independent active free play in children. | Children | SR and ‘meta-study’; 46 studies | Perceived facilitators include sex (male), competence, safety, neighbourhood (e.g., number of children), playground quality (e.g., need for maintenance), and ‘good parenting ideals’. |
| 47 | Lee, Pope (46) | Review the impact of youth sport participation on children’s PA | Children and adolescents aged ≤19 years | SR; 44 studies | Greater amounts and frequency of engagement in organized youth sport were associated with a more physically active lifestyle in youth and/or later in adulthood. |
| 48 | Lima-Serrano and Lima-Rodriguez (47) | Summarise the characteristics and effects of school-based interventions acting on different behavioural domains of adolescent health promotion | Adolescents | SR; 35 studies | Most interventions aimed at promoting health nutrition and PA found moderate to large effects. No comments on study specifics/ Moderators. |
| 49 | Lindsay, Greaney (48) | Synthesise evidence for on influences on PA in pre-school aged children | Preschool children | SR; 158 studies | Positive association for family income, sex (male), siblings, ethnicity (Caucasian), parent influence, safety, weather (summer, spring), and childcare. No association for parenting style and PA identified. Policy influence unclear. |
| 50 | Loprinzi, Davis (49) | To propose a model connecting motor skill development and child PA | Children and adolescents | Narrative review | Motor skills are a positive influence on PA. Narrative review only. |
| 51 | Lu, McKyer (50) | Critically assess perceived barriers to children’s Active Commuting to School | School aged children | SR; 39 studies | Barriers included personal/ family (e.g., time, preference), environment (e.g., safety, distance), and social (e.g., neighbourhood safety). |
| 52 | Maher, Lewis (51) | Review evidence regarding the effectiveness of online social network health behaviour interventions | Children | SR; 10 studies | Of the four studies that investigated PA behaviour change, one had legible effect, two moderate and one large effects. |
| 53 | Maitland, Stratton (52) | Review the influence of the home physical environment on the sedentary behaviour and PA. | Aged 8 – 14 years | SR; 49 studies | Limited and inconsistent associations between media equipment in the home and PA outcomes. PA equipment, yard space, and social support were positively associated with PA. Living in an apartment was negatively associated with PA. |
| 54 | Martin and Murtagh (53) | Review classroom-based PA interventions that integrate academic content and assess the effectiveness of the interventions on PA and other health outcomes | School aged children | SR; 15 studies | Positive medium to large effects in more than half the studies examined. |
| 55 | Martins, Marques (54) | Examine qualitative studies of adolescents’ perspectives about the facilitators and barriers of PA. | Adolescents | SR; 12 | Barriers included competition and motivational climate, attitude toward PA (especially in inactive girls), perceptions ofr femininity/ gender roles, time, and low perception of competence. Facilitators included family support, value of health benefits, intrinsic motivation, and mastery motivation, and having fun. Values varied by activity level. |
| 56 | Maturo and Cunningham (55) | Determine the role of friends in shaping PA among children and adolescents. | Aged < 19 years of attending secondary school or lower. | SR; 81 studies | Friendship positively influences PA. Three broad mechanisms including communication/ support, modelling, and participation. |
| 57 | McCrorie, Fenton (56) | Review studies that have combined GPS, GIS and accelerometry to investigate the PA-environment relationship. | Aged 5 – 18 years | SR; 14 studies | Access to greenspace and time spent outdoors positively associated with PA. |
| 58 | McGrath, Hopkins (57) | Examine the environment – PA relationship in youth. | Children and adolescents | SR and MA; 23 studies | Play space (trivial to small effects), walkability (trivial to moderate effects), and safety (small to moderate effects) influenced PA. Effects were modified by age. |
| 59 | McIntosh, Jay (58) | Assess the effectiveness of E-health interventions in increasing PA | School or university aged children and young adults | SR; 10 studies | Most studies increased PA. Interventions without theory did not increase PA. |
| 60 | Mehtala, Saakslahti (59) | Review the evidence on PA interventions in childcare by applying a socio-ecological approach. | Children aged 2 – 6 years | SR; 23 studies | Increases in PA were achieved in most but not all studies. Non-theory-based interventions were more likely to increase PA. The most effective interventions targeted personal characteristics or teacher training. |
| 61 | Mendonca, Cheng (60) | Synthesise evidence for social support and PA. | Adolescents | SR; 75 studies | Social support from friends and family but not teachers positively associated with PA. |
| 62 | Morton, Atkin (61) | Summarise evidence for school-based policy, physical and social-environmental influences on adolescent PA. | Adolescents | SR; 68 (qualitative) and 25 (quantitative) studies | Availability/ accessibility of facilities, as well as social environment are important. Indirect influence of school policy via its effects on school culture, active travel, uniforms, break time, and activity rules. |
| 63 | Nasuti and Rhodes (62) | Determine the relationship between affective judgment and PA in youth | Aged 5 – 18 years | SR and MA; 55 studies. | Affective judgement positively influences PA (medium effect size). Moderated by gender/ sex. Single component affective judgement interventions show some ability to increase PA. |
| 64 | Nathan, Elton (63) | Describe factors that influence school PA policy implementation | School children | SR; 9 studies | Barriers and facilitators include competing interests/ goals, environment and resources, social influences, and beliefs about capabilities. |
| 65 | Naylor, Nettlefold (64) | Establish the evidence related to implementation of school-based PA models to explore the relationship between implementation and health outcomes and factors that influence implementation. | School aged children | SR; 38 studies | Time, quality/availability of resources, supportive school climate, contextual appropriateness of the intervention, availability of training and teacher self-efficacy influenced implementation. |
| 66 | Neshteruk, Nezami (65) | Synthesise observational evidence for the influence of fathers on children's PA. | Children | SR; 10 studies | Positive influence of fathers on child PA but low availability of evidence. |
| 67 | Nguyen, Hacker (66) | Analyse contributors to success of a PA interventions | School aged children | SR; 50 studies | Mixed but generally more positive effects of interventions on PA. Behavioural and social approaches, information approaches, and environment approaches can, but do not always, increase PA. Environment most consistent effect. |
| 68 | Norris, Shelton (67) | Investigate methods used in active lessons and education interventions and their effects on PA and educational outcomes. | Children and adolescents | SR; 11 studies | Active lessons increased PA. |
| 69 | Norris, Hamer (68) | Assess the quality of evidence for the effects of school active video game on PA and health | School aged children | SR; 22 studies | Some but not all interventions increased light PA and decreased sedentary time but the quality was low and conclusions limited. |
| 70 | O'Donoghue, Kennedy (69) | Provide an overview evidence for socioeconomic correlates and determinants of PA. | Entire lifespan | Umbrella review; 19 reviews | Socioeconomic status was unrelated to PA. Limited evidence for a positive association between parent education or income and childhood PA. |
| 71 | Oglund, Hildebrand (70) | Examine the evidence for birth weight, early growth and motor development as determinants of PA. | Children | SR; 11 studies | Birthweight not associated with PA. Early growth negatively influences PA. Inconsistent findings for early motor development and PA. |
| 72 | Oglund, Hildebrand (70) | To summarize the existing knowledge on early life determinants  of childhood PA  and sedentary time, | Infants | SR and MA; 9 studies | There is insufficient evidence to conclude whether any of the included early life factors predicts childhood PA and sedentary time |
| 73 | Oliveira, Moreira (71) | Examine environmental determinants of PA | Children | SR; 28 studies | Proximity of parks and playgrounds, safety perceptions, low traffic, and the presence of sidewalks and bike lanes were positively associated with PA. |
| 74 | Olstad, Ancilotto (72) | Synthesise evidence for policy impact on PA and other health outcomes | Children and adults | SR; 18 studies | Provision of information by the government may result in an increase in PA. Changes in built environment less so. |
| 75 | Owen, Curry (73) | Assess the impact and design of school-based PA interventions targeting adolescent girls. | Adolescent girls | SR and MA; 20 studies | Small positive increases in PA following intervention. Multi-component interventions and interventions underpinned by theory had more positive results. |
| 76 | K, Smith (74) | Examine the association between self-determined motivation and PA | Children and adolescents | SR and MA; 46 studies | Motivation predicted PA. |
| 77 | Parisod, Pakarinen (75) | Determine whether active video games improve child health measures | Children and adolescents | Umbrella review; 15 reviews | Active video games provide light to moderate PA but unclear whether this helps children meet PA recommendations. No comment on PA change. |
| 78 | Parrish, Okely (76) | Examine the effects of recess-based interventions on the PA | School aged children and adolescents | SR; 9 studies | Playground marking and equipment as well as combined interventions can increase PA but overall evidence limited. |
| 79 | Pearson, Braithwaite (77) | Quantify effectiveness of PA interventions in girls | Adolescent girls. | SR and MA; 45 studies | Small but significant increase in PA overall. High heterogeneity in results. Multi-component interventions, theory-based interventions, and interventions delivered to just girls more likely to see increases. |
| 80 | Plotnikoff, Costigan (78) | Review and examine the explanatory power of key social-cognitive theories used to explain PA intention and behaviour | Adolescents | SR and MA; 23 studies | Social cognitive models explained 48% and 33% of the variance in PA intention and PA behaviour, respectively. |
| 81 | Puggina, Aleksovska (79) | Summarise evidence for policy determinants of PA | Entire lifespan | Umbrella review; 14 studies | Policies included for children and adolescents included home, school level policies. Home level policies had mixed influence on PA. School level policies had a positive influence on PA. Levels of evidence mixed. |
| 82 | Quitério (80) | Assess the effectiveness of physical education interventions for health | School aged children | SR; 27 studies | PE interventions can increase PA. An absence of critical analysis makes the findings difficult to interpret. |
| 83 | Riso, Kull (81) | Determine whether school-based interventions influence PA level and describe the main components of PA interventions. | Children aged 6 – 12 years | SR; 17 studies | PA increases were achieved in several interventions. Effective school-based intervention models include additional PE lessons, active recess, activity breaks, and changes in school environments. |
| 84 | Robertson-Wilson, Reinders (82) | Assess the effectiveness of dance interventions on child PA | Children and adolescents. | SR; 13 studies | Inconsistent findings for the ability of dance interventions to increase PA. |
| 85 | Rose, Barker (83) | Synthesise the evidence for the effectiveness of digital interventions to improve diet quality and increase PA | Adolescents | SR; 27 studies | Inconsistent findings for increases in PA. More effective interventions included education, goal setting, and self-monitoring paired with goal setting. |
| 86 | Saitta, Devan (84) | Synthesis of the evidence for park-based PA interventions for persons with disabilities | People with disabilities | SR; 10 studies | No evidence for change in PA with park environment accessibility for disabilities but very limited studies and evidence quality |
| 87 | Smith, Norgate (85) | To examine association between walking school buses and PA | School aged children | SR; 12 studies | Walking school buses increase PA, although the increase is not always significant. |
| 88 | Spencer, Rehman (86) | Examine how gender norms are understood in relation to PA and nutrition in young girls. | Female children and adolescents. | Scoping review | Young girls can enjoy and benefit from PA; however, pressure to appear feminine can limit participation and engagement. |
| 89 | Sterdt, Liersch (87) | Identify promoting and inhibiting correlates associated with PA | Aged 3 – 18 years. | Umbrella review; 10 reviews | Consistent positive associations between sex (male), socioeconomic status, access to parks, time outdoors, school policy, and PA. Inconsistent associations for age, body mass, ethnicity, and sedentary time. Negative associations for perceived barriers, mental health road safety concerns and PA. |
| 90 | Temple and Robinson (88) | Review of school-based interventions to promote PA | Pre-school children | SR; 14 studies | Manipulating the playground, markings, equipment, number of children playing, goal setting, and reinforcement were associated with increases in PA. |
| 91 | Timperio, Reid (89) | Examine evidence for the built and social environment on PA. | Children | SR; 26 studies | Recent evidence for the neighbourhood built and social environment on children’s PA is modest. Few neighbourhood attributes were consistently associated with children’s PA in the expected direction. |
| 92 | Tonge, Jones (90) | Review the correlates of PA and sedentary behaviour among children | Children  in early childhood education and care services | SR; 27 studies | Sex (male), age, motor control, and outdoor environments positively associated with PA. Educator and policy factors unclear. |
| 93 | Van Hecke, Ghekiere (91) | Examine characteristics of public open spaces and PA | Adolescents. | SR; 31 studies | Availability facilities, design, safety, cleanliness, and policy (e.g., restrictive rules) perceived to influence adolescent engagement and PA. |
| 94 | Villa-Gonzalez, Barranco-Ruiz (92) | Examine the effectiveness of interventions focused on active travel to school | School aged children | SR; 23 studies | Active school transport increased in several studies by varying amounts. Overall, the evidence is limited. |
| 95 | Voskuil, Frambes (93) | Evaluate evidence for PA intervention effects on accelerometer-measured PA, body mass index, and percent body fat percentage | School aged girls | SR; 15 studies | Limited effectiveness for the interventions studied. |
| 96 | Ward, Belanger (94) & | Examine influence of childcare educators’ PA and preschool children | Preschool children | SR; 15 studies | Portable play equipment, child initiative contribute to PA. |
| 97 | Ward, Belanger (95) | Examine how peers’ behaviours impact preschool PA | Preschool children | SR; 13 studies | Number of peers, and familiarity contribute to PA. Weak evidence overall. |
| 98 | Watson, Timperio (96) | Examine benefit of classroom-based PA interventions on PA and academic outcomes. | School aged children | SR and MA; 39 studies | A 2% to 16% increase in MVPA during intervention lessons and a 2% to 12% increase in school day MVPA in all studies. |
| 99 | Weatherson, Gainforth (97) | Summarize the implementation status approaches used to implement daily PA and the effectiveness interventions for increasing PA | School aged children | Scoping review; 15 studies | Methods have included prescriptive and non-prescriptive approaches. Common barriers and facilitators of implementation include environmental context and resources, beliefs about consequences, and social influences. Students that receive PA or PE class more likely to meet MVPA guidelines but limited evidence overall. |
| 100 | Xu, Wen (98) | Examine parental influences on PA and screen time | Children | SR; 30 studies | Parents influence PA most via encouragement and support. |
| 101 | Yao and Rhodes (99) | Examine parental correlates in child and adolescent PA | Children and adolescents | SR and MA; 112 studies | Parental modelling and support have small to moderate effects on child PA. |

SR – Systematic Review, MA – Meta Analyses, NR – Narrative review, PA – Physical Activity, PE – Physical Education MVPA – Moderate- to Vigorous-intensity Physical Activity, BMI – Body Mass Index

**References**

1. An R, Yang Y, Hoschke A, Xue H, Wang Y. Influence of neighbourhood safety on childhood obesity: a systematic review and meta-analysis of longitudinal studies. Obes Rev. 2017;18(11):1289-309.

2. Aura A, Sormunen M, Tossavainen K. The relation of socio-ecological factors to adolescents’ health-related behaviour. Health Education. 2016;116(2):177-201.

3. Ball K, Carver A, Downing K, Jackson M, O'Rourke K. Addressing the social determinants of inequities in physical activity and sedentary behaviours. Health Promot Int. 2015;30 Suppl 2:ii18-9.

4. Biddle SJ, Braithwaite R, Pearson N. The effectiveness of interventions to increase physical activity among young girls: a meta-analysis. Preventive medicine. 2014;62:119-31.

5. Bonell C, Wells H, Harden A, Jamal F, Fletcher A, Thomas J, et al. The effects on student health of interventions modifying the school environment: systematic review. J Epidemiol Community Health. 2013;67(8):677-81.

6. Borde R, Smith JJ, Sutherland R, Nathan N, Lubans DR. Methodological considerations and impact of school-based interventions on objectively measured physical activity in adolescents: a systematic review and meta-analysis. Obes Rev. 2017;18(4):476-90.

7. Brown H, Hume C, Pearson N, Salmon J. A systematic review of intervention effects on potential mediators of children's physical activity. BMC public health. 2013;13:165.

8. Brown HE, Atkin AJ, Panter J, Wong G, Chinapaw MJ, van Sluijs EM. Family-based interventions to increase physical activity in children: a systematic review, meta-analysis and realist synthesis. Obes Rev. 2016;17(4):345-60.

9. Bush PL, Garcia Bengoechea E. What do we know about how to promote physical activity to adolescents? A mapping review. Health Educ Res. 2015;30(5):756-72.

10. Carlin A, Murphy MH, Gallagher AM. Do Interventions to Increase Walking Work? A Systematic Review of Interventions in Children and Adolescents. Sports medicine (Auckland, NZ). 2016;46(4):515-30.

11. Cerruti Dellert J, Johnson P. Interventions With Children and Parents to Improve Physical Activity and Body Mass Index: A Meta- analysis. American Journal of Health Promotion. 2014;28(4):259-67.

12. Chung SJ, Ersig AL, McCarthy AM. The Influence of Peers on Diet and Exercise Among Adolescents: A Systematic Review. J Pediatr Nurs. 2017;36:44-56.

13. Condello G, Puggina A, Aleksovska K, Buck C, Burns C, Cardon G, et al. Behavioral determinants of physical activity across the life course: a "DEterminants of DIet and Physical ACtivity" (DEDIPAC) umbrella systematic literature review. The international journal of behavioral nutrition and physical activity. 2017;14(1):58.

14. Corepal R, Tully MA, Kee F, Miller SJ, Hunter RF. Behavioural incentive interventions for health behaviour change in young people (5-18years old): A systematic review and meta-analysis. Preventive medicine. 2018;110:55-66.

15. Cortis C, Puggina A, Pesce C, Aleksovska K, Buck C, Burns C, et al. Psychological determinants of physical activity across the life course: A "DEterminants of DIet and Physical ACtivity" (DEDIPAC) umbrella systematic literature review. PloS one. 2017;12(8):e0182709.

16. Craike M, Wiesner G, Hilland TA, Bengoechea EG. Interventions to improve physical activity among socioeconomically disadvantaged groups: an umbrella review. The international journal of behavioral nutrition and physical activity. 2018;15(1):43.

17. Crawford SK, Stafford KN, Phillips SM, Scott KJ, Tucker P. Strategies for Inclusion in Play among Children with Physical Disabilities in Childcare Centers: An Integrative Review. Physical & Occupational Therapy In Pediatrics. 2014;34(4):404-23.

18. Cushing CC, Brannon EE, Suorsa KI, Wilson DK. Systematic review and meta-analysis of health promotion interventions for children and adolescents using an ecological framework. J Pediatr Psychol. 2014;39(8):949-62.

19. Direito A, Carraca E, Rawstorn J, Whittaker R, Maddison R. mHealth Technologies to Influence Physical Activity and Sedentary Behaviors: Behavior Change Techniques, Systematic Review and Meta-Analysis of Randomized Controlled Trials. Ann Behav Med. 2017;51(2):226-39.

20. Dobbins M, Husson H, DeCorby K, LaRocca RL. School-based physical activity programs for promoting physical activity and fitness in children and adolescents aged 6 to 18. Cochrane Database Syst Rev. 2013;2013(2):CD007651.

21. Draper CE, Grobler L, Micklesfield LK, Norris SA. Impact of social norms and social support on diet, physical activity and sedentary behaviour of adolescents: a scoping review. Child Care Health Dev. 2015;41(5):654-67.

22. Errisuriz VL, Golaszewski NM, Born K, Bartholomew JB. Systematic Review of Physical Education-Based Physical Activity Interventions Among Elementary School Children. J Prim Prev. 2018;39(3):303-27.

23. Erwin HE, Ickes M, Ahn S, Fedewa A. Impact of recess interventions on children's physical activity--a meta-analysis. Am J Health Promot. 2014;28(3):159-67.

24. Escalante Y, Garcia-Hermoso A, Backx K, Saavedra JM. Playground designs to increase physical activity levels during school recess: a systematic review. Health Educ Behav. 2014;41(2):138-44.

25. Figueroa R, An R. Motor Skill Competence and Physical Activity in Preschoolers: A Review. Matern Child Health J. 2017;21(1):136-46.

26. Fisher A, Smith L, van Jaarsveld CH, Sawyer A, Wardle J. Are children's activity levels determined by their genes or environment? A systematic review of twin studies. Prev Med Rep. 2015;2:548-53.

27. Gordon ES, Tucker P, Burke SM, Carron AV. Effectiveness of physical activity interventions for preschoolers: a meta-analysis. Research quarterly for exercise and sport. 2013;84(3):287-94.

28. Herring MP, Sailors MH, Bray MS. Genetic factors in exercise adoption, adherence and obesity. Obes Rev. 2014;15(1):29-39.

29. Hesketh KR, O'Malley C, Paes VM, Moore H, Summerbell C, Ong KK, et al. Determinants of Change in Physical Activity in Children 0-6 years of Age: A Systematic Review of Quantitative Literature. Sports medicine (Auckland, NZ). 2017;47(7):1349-74.

30. Hesketh KR, Lakshman R, van Sluijs EMF. Barriers and facilitators to young children's physical activity and sedentary behaviour: a systematic review and synthesis of qualitative literature. Obes Rev. 2017;18(9):987-1017.

31. Hieftje K, Edelman EJ, Camenga DR, Fiellin LE. Electronic media-based health interventions promoting behavior change in youth: a systematic review. JAMA Pediatr. 2013;167(6):574-80.

32. Hodges EA, Smith C, Tidwell S, Berry D. Promoting physical activity in preschoolers to prevent obesity: a review of the literature. J Pediatr Nurs. 2013;28(1):3-19.

33. Hynynen ST, van Stralen MM, Sniehotta FF, Araújo-Soares V, Hardeman W, Chinapaw MJM, et al. A systematic review of school-based interventions targeting physical activity and sedentary behaviour among older adolescents. International Review of Sport & Exercise Psychology. 2016;9(1):22-44.

34. Jaeschke L, Steinbrecher A, Luzak A, Puggina A, Aleksovska K, Buck C, et al. Socio-cultural determinants of physical activity across the life course: a 'Determinants of Diet and Physical Activity' (DEDIPAC) umbrella systematic literature review. Int J Behav Nutr Phys Act. 2017;14(1):173.

35. Jiying L, Robbins LB, Fujun W, Wei P. Interventions to Increase Physical Activity in Children Aged 2-5 Years: A Systematic Review. Pediatric Exercise Science. 2015;27(3):314-33.

36. Johnstone A, Hughes AR, Martin A, Reilly JJ. Utilising active play interventions to promote physical activity and improve fundamental movement skills in children: a systematic review and meta-analysis. BMC public health. 2018;18(1):789.

37. Kader M, Sundblom E, Elinder LS. Effectiveness of universal parental support interventions addressing children's dietary habits, physical activity and bodyweight: A systematic review. Preventive medicine. 2015;77:52-67.

38. Kaushal N, Rhodes RE. The home physical environment and its relationship with physical activity and sedentary behavior: a systematic review. Preventive medicine. 2014;67:221-37.

39. Lai S, Costigan S, Morgan P, Lubans D, Stodden D, Salmon J, et al. Do School-Based Interventions Focusing on Physical Activity, Fitness, or Fundamental Movement Skill Competency Produce a Sustained Impact in These Outcomes in Children and Adolescents? A Systematic Review of Follow-Up Studies. Sports Medicine. 2014;44(1):67-79.

40. Laird Y, Fawkner S, Kelly P, McNamee L, Niven A. The role of social support on physical activity behaviour in adolescent girls: a systematic review and meta-analysis. The international journal of behavioral nutrition and physical activity. 2016;13(1):79.

41. Langford R, Bonell C, Jones H, Pouliou T, Murphy S, Waters E, et al. The World Health Organization's Health Promoting Schools framework: a Cochrane systematic review and meta-analysis. BMC Public Health. 2015;15:130.

42. Larouche R. Built Environment Features that Promote Cycling in School-Aged Children. Curr Obes Rep. 2015;4(4):494-503.

43. Larouche R, Mammen G, Rowe DA, Faulkner G. Effectiveness of active school transport interventions: a systematic review and update. BMC public health. 2018;18(1):206.

44. LeBlanc AG, Chaput JP, McFarlane A, Colley RC, Thivel D, Biddle SJ, et al. Active video games and health indicators in children and youth: a systematic review. PloS one. 2013;8(6):e65351.

45. Lee H, Tamminen KA, Clark AM, Slater L, Spence JC, Holt NL. A meta-study of qualitative research examining determinants of children's independent active free play. The international journal of behavioral nutrition and physical activity. 2015;12(1):5.

46. Lee JE, Pope Z, Gao Z. The Role of Youth Sports in Promoting Children's Physical Activity and Preventing Pediatric Obesity: A Systematic Review. Behav Med. 2018;44(1):62-76.

47. Lima-Serrano M, Lima-Rodriguez JS. Impact of school-based health promotion interventions aimed at different behavioral domains: a systematic review. Gac Sanit. 2014;28(5):411-7.

48. Lindsay AC, Greaney ML, Wallington SF, Mesa T, Salas CF. A review of early influences on physical activity and sedentary behaviors of preschool-age children in high-income countries. J Spec Pediatr Nurs. 2017;22(3).

49. Loprinzi PD, Davis RE, Fu YC. Early motor skill competence as a mediator of child and adult physical activity. Prev Med Rep. 2015;2:833-8.

50. Lu W, McKyer EL, Lee C, Goodson P, Ory MG, Wang S. Perceived barriers to children's active commuting to school: a systematic review of empirical, methodological and theoretical evidence. The international journal of behavioral nutrition and physical activity. 2014;11(1):140.

51. Maher CA, Lewis LK, Ferrar K, Marshall S, De Bourdeaudhuij I, Vandelanotte C. Are health behavior change interventions that use online social networks effective? A systematic review. J Med Internet Res. 2014;16(2):e40.

52. Maitland C, Stratton G, Foster S, Braham R, Rosenberg M. A place for play? The influence of the home physical environment on children's physical activity and sedentary behaviour. The international journal of behavioral nutrition and physical activity. 2013;10:99.

53. Martin R, Murtagh EM. Effect of Active Lessons on Physical Activity, Academic, and Health Outcomes: A Systematic Review. Research quarterly for exercise and sport. 2017;88(2):149-68.

54. Martins J, Marques A, Sarmento H, Carreiro da Costa F. Adolescents' perspectives on the barriers and facilitators of physical activity: a systematic review of qualitative studies. Health education research. 2015;30(5):742-55.

55. Maturo CC, Cunningham SA. Influence of friends on children's physical activity: a review. American journal of public health. 2013;103(7):e23-38.

56. McCrorie PR, Fenton C, Ellaway A. Combining GPS, GIS, and accelerometry to explore the physical activity and environment relationship in children and young people - a review. The international journal of behavioral nutrition and physical activity. 2014;11(1):93.

57. McGrath LJ, Hopkins WG, Hinckson EA. Associations of objectively measured built-environment attributes with youth moderate-vigorous physical activity: a systematic review and meta-analysis. Sports medicine (Auckland, NZ). 2015;45(6):841-65.

58. McIntosh JRD, Jay S, Hadden N, Whittaker PJ. Do E-health interventions improve physical activity in young people: a systematic review. Public Health. 2017;148:140-8.

59. Mehtala MA, Saakslahti AK, Inkinen ME, Poskiparta ME. A socio-ecological approach to physical activity interventions in childcare: a systematic review. The international journal of behavioral nutrition and physical activity. 2014;11(1):22.

60. Mendonca G, Cheng LA, Melo EN, de Farias Junior JC. Physical activity and social support in adolescents: a systematic review. Health Educ Res. 2014;29(5):822-39.

61. Morton KL, Atkin AJ, Corder K, Suhrcke M, van Sluijs EM. The school environment and adolescent physical activity and sedentary behaviour: a mixed-studies systematic review. Obes Rev. 2016;17(2):142-58.

62. Nasuti G, Rhodes RE. Affective judgment and physical activity in youth: review and meta-analyses. Ann Behav Med. 2013;45(3):357-76.

63. Nathan N, Elton B, Babic M, McCarthy N, Sutherland R, Presseau J, et al. Barriers and facilitators to the implementation of physical activity policies in schools: A systematic review. Preventive medicine. 2018;107:45-53.

64. Naylor PJ, Nettlefold L, Race D, Hoy C, Ashe MC, Wharf Higgins J, et al. Implementation of school based physical activity interventions: a systematic review. Preventive medicine. 2015;72:95-115.

65. Neshteruk CD, Nezami BT, Nino-Tapias G, Davison KK, Ward DS. The influence of fathers on children's physical activity: A review of the literature from 2009 to 2015. Preventive medicine. 2017;102:12-9.

66. Nguyen S, Hacker AL, Henderson M, Barnett T, Mathieu ME, Pagani L, et al. Physical Activity Programs with Post-Intervention Follow-Up in Children: A Comprehensive Review According to Categories of Intervention. International journal of environmental research and public health. 2016;13(7):30.

67. Norris E, Shelton N, Dunsmuir S, Duke-Williams O, Stamatakis E. Physically active lessons as physical activity and educational interventions: a systematic review of methods and results. Preventive medicine. 2015;72:116-25.

68. Norris E, Hamer M, Stamatakis E. Active Video Games in Schools and Effects on Physical Activity and Health: A Systematic Review. J Pediatr. 2016;172:40-6 e5.

69. O'Donoghue G, Kennedy A, Puggina A, Aleksovska K, Buck C, Burns C, et al. Socio-economic determinants of physical activity across the life course: A "DEterminants of DIet and Physical ACtivity" (DEDIPAC) umbrella literature review. PloS one. 2018;13(1):e0190737.

70. Oglund GP, Hildebrand M, Ekelund U. Are Birth Weight, Early Growth, and Motor Development Determinants of Physical Activity in Children and Youth? A Systematic Review and Meta-Analysis. Pediatr Exerc Sci. 2015;27(4):441-53.

71. Oliveira AF, Moreira C, Abreu S, Mota J, Santos R. Environmental determinants of physical activity in children: A systematic review. Archives of Exercise in Health & Disease. 2014;4(2):254-61.

72. Olstad DL, Ancilotto R, Teychenne M, Minaker LM, Taber DR, Raine KD, et al. Can targeted policies reduce obesity and improve obesity-related behaviours in socioeconomically disadvantaged populations? A systematic review. Obesity Reviews. 2017;18(7):791-807.

73. Owen MB, Curry WB, Kerner C, Newson L, Fairclough SJ. The effectiveness of school-based physical activity interventions for adolescent girls: A systematic review and meta-analysis. Preventive medicine. 2017;105:237-49.

74. K BO, Smith J, Lubans DR, Ng JY, Lonsdale C. Self-determined motivation and physical activity in children and adolescents: a systematic review and meta-analysis. Prev Med. 2014;67:270-9.

75. Parisod H, Pakarinen A, Kauhanen L, Aromaa M, Leppanen V, Liukkonen TN, et al. Promoting Children's Health with Digital Games: A Review of Reviews. Games Health J. 2014;3(3):145-56.

76. Parrish AM, Okely AD, Stanley RM, Ridgers ND. The effect of school recess interventions on physical activity : a systematic review. Sports medicine (Auckland, NZ). 2013;43(4):287-99.

77. Pearson N, Braithwaite R, Biddle SJ. The effectiveness of interventions to increase physical activity among adolescent girls: a meta-analysis. Acad Pediatr. 2015;15(1):9-18.

78. Plotnikoff RC, Costigan SA, Karunamuni N, Lubans DR. Social cognitive theories used to explain physical activity behavior in adolescents: a systematic review and meta-analysis. Preventive medicine. 2013;56(5):245-53.

79. Puggina A, Aleksovska K, Buck C, Burns C, Cardon G, Carlin A, et al. Policy determinants of physical activity across the life course: a 'DEDIPAC' umbrella systematic literature review. Eur J Public Health. 2018;28(1):105-18.

80. Quitério ALD. School physical education: The effectiveness of health-related interventions and recommendations for health-promotion practice. Health Education Journal. 2012;72(6):716-32.

81. Riso E-M, Kull M, Hannus A. OBJECTIVELY MEASURED SCHOOL-BASED PHYSICAL ACTIVITY INTERVENTIONS FOR 6-12-YEAR-OLD CHILDREN IN 2009-2014: A SYSTEMATIC REVIEW. Acta Kinesiologiae Universitatis Tartuensis. 2014;20:9-24.

82. Robertson-Wilson J, Reinders N, Bryden PJ. Dance Interventions to Increase Physical Activity Among Youth: A Systematic Review. Kinesiology Review. 2016;5(3):170-88.

83. Rose T, Barker M, Maria Jacob C, Morrison L, Lawrence W, Strommer S, et al. A Systematic Review of Digital Interventions for Improving the Diet and Physical Activity Behaviors of Adolescents. J Adolesc Health. 2017;61(6):669-77.

84. Saitta M, Devan H, Boland P, Perry MA. Park-based physical activity interventions for persons with disabilities: A mixed-methods systematic review. Disabil Health J. 2019;12(1):11-23.

85. Smith L, Norgate SH, Cherrett T, Davies N, Winstanley C, Harding M. Walking school buses as a form of active transportation for children-a review of the evidence. J Sch Health. 2015;85(3):197-210.

86. Spencer RA, Rehman L, Kirk SF. Understanding gender norms, nutrition, and physical activity in adolescent girls: a scoping review. The international journal of behavioral nutrition and physical activity. 2015;12(1):6.

87. Sterdt E, Liersch S, Walter U. Correlates of physical activity of children and adolescents: A systematic review of reviews. Health Education Journal. 2013;73(1):72-89.

88. Temple M, Robinson JC. A systematic review of interventions to promote physical activity in the preschool setting. J Spec Pediatr Nurs. 2014;19(4):274-84.

89. Timperio A, Reid J, Veitch J. Playability: Built and Social Environment Features That Promote Physical Activity Within Children. Curr Obes Rep. 2015;4(4):460-76.

90. Tonge KL, Jones RA, Okely AD. Correlates of children's objectively measured physical activity and sedentary behavior in early childhood education and care services: A systematic review. Preventive medicine. 2016;89:129-39.

91. Van Hecke L, Ghekiere A, Veitch J, Van Dyck D, Van Cauwenberg J, Clarys P, et al. Public open space characteristics influencing adolescents' use and physical activity: A systematic literature review of qualitative and quantitative studies. Health & place. 2018;51:158-73.

92. Villa-Gonzalez E, Barranco-Ruiz Y, Evenson KR, Chillon P. Systematic review of interventions for promoting active school transport. Preventive medicine. 2018;111:115-34.

93. Voskuil VR, Frambes DA, Robbins LB. Effect of Physical Activity Interventions for Girls on Objectively Measured Outcomes: A Systematic Review of Randomized Controlled Trials. J Pediatr Health Care. 2017;31(1):75-87.

94. Ward S, Belanger M, Donovan D, Carrier N. Systematic review of the relationship between childcare educators' practices and preschoolers' physical activity and eating behaviours. Obes Rev. 2015;16(12):1055-70.

95. Ward SA, Belanger MF, Donovan D, Carrier N. Relationship between eating behaviors and physical activity of preschoolers and their peers: a systematic review. The international journal of behavioral nutrition and physical activity. 2016;13:50.

96. Watson A, Timperio A, Brown H, Best K, Hesketh KD. Effect of classroom-based physical activity interventions on academic and physical activity outcomes: a systematic review and meta-analysis. The international journal of behavioral nutrition and physical activity. 2017;14(1):114.

97. Weatherson KA, Gainforth HL, Jung ME. A theoretical analysis of the barriers and facilitators to the implementation of school-based physical activity policies in Canada: a mixed methods scoping review. Implement Sci. 2017;12(1):41.

98. Xu H, Wen LM, Rissel C. Associations of parental influences with physical activity and screen time among young children: a systematic review. Journal of obesity. 2015;2015:546925.

99. Yao CA, Rhodes RE. Parental correlates in child and adolescent physical activity: a meta-analysis. The international journal of behavioral nutrition and physical activity. 2015;12(1):10.
